# Progression of brain injuries associated with methotrexate chemotherapy in childhood acute lymphoblastic leukemia

**DOI:** 10.1038/s41390-024-03351-9

**Published:** 2024-07-01

**Authors:** Ravi Bansal, Deepa Bhojwani, Bernice F. Sun, Siddhant Sawardekar, Alan S. Wayne, Hannah Ouassil, Chaitanya Gupte, Courtney Marcelino, Maria J. Gonzalez Anaya, Natalia Luna, Bradley S. Peterson

**Affiliations:** 1 Department of Psychiatry, Keck School of Medicine, University of Southern California, Los Angeles, CA USA; 2 Institute for the Developing Mind, Children’s Hospital Los Angeles, Los Angeles, CA USA; 3 Cancer and Blood Disease Institute, Children’s Hospital Los Angeles, Norris Comprehensive Cancer Center and Keck School of Medicine, University of Southern California, Los Angeles, CA USA; 4 College of Letters, Arts and Sciences, University of Southern California, Los Angeles, CA USA

## Abstract

**Background:**

Brain bases and progression of methotrexate-associated neurotoxicity and cognitive disturbances remain unknown. We tested whether brain abnormalities worsen in proportion to intrathecal methotrexate(IT-MTX) doses.

**Methods:**

In this prospective, longitudinal study, we recruited 19 patients with newly diagnosed acute lymphoblastic leukemia 4-to-20 years of age and 20 matched controls. We collected MRI and neuropsychological assessments at a pre-methotrexate baseline and at week 9, week 22, and year 1 during treatment.

**Results:**

Patients had baseline abnormalities in cortical and subcortical gray matter(GM), white matter(WM) volumes and microstructure, regional cerebral blood flow, and neuronal density. Abnormalities of GM, blood flow, and metabolites worsened in direct proportions to IT-MTX doses. WM abnormalities persisted until week 22 but normalized by year 1. Brain injuries were localized to dorsal and ventral attentional and frontoparietal cognitive networks. Patients had cognitive deficits at baseline that persisted at 1-year follow-up.

**Conclusions:**

Baseline abnormalities are likely a consequence of neuroinflammation and oxidative stress. Baseline abnormalities in WM microstructure and volumes, and blood flow persisted until week 22 but normalized by year 1, likely due to treatment and its effects on reducing inflammation. The cytotoxic effects of IT-MTX, however, likely contributed to continued, progressive cortical thinning and reductions in neuronal density, thereby contributing to enduring cognitive deficits.

**Impact:**

- Brain abnormalities at a pre-methotrexate baseline likely are due to acute illness. The cytotoxic effects of intrathecal MTX contribute to progressive cortical thinning, reductions in neuronal density, and enduring cognitive deficits.
- Baseline white matter abnormalities may have normalized via methotrexate treatment and decreasing neuroinflammation. Corticosteroid and leucovorin conferred neuroprotective effects.
- Our findings suggest that the administration of neuroprotective and anti-inflammatory agents should be considered even earlier than they are currently administered. The neuroprotective effects of leucovorin suggest that strategies may be developed that extend the duration of this intervention or adapt it for use in standard risk patients.

## Introduction

Acute lymphoblastic leukemia (ALL) is the most prevalent childhood cancer. Its treatment involves multiagent chemotherapy,^1^ with methotrexate (MTX) being the essential core component, particularly for prophylaxis and treatment of CNS involvement. MTX, however, is associated with the development of cognitive,^2,3^ behavioral, and emotional disturbances.^1,4^ These effects can persist after treatment, as ALL survivors are reported to have lower measures of IQ, working memory, attention, and information processing speed.^5–8^ Cognitive deficits likely derive from either illness- or treatment-related injury to the brain, as MRI studies have associated MTX with leukoencephalopathy,^1,9^ white matter (WM) hyperintensities,^1,10–12^ altered WM microstructure,^2,13,14^ and reduced WM volumes,^15,16^ glymphatic system abnormalities,^17^ atrophy of cortical and subcortical gray matter,^4,18–21^ and altered brain activity.^22^

Whether abnormalities in brain structure and function are consequences of MTX chemotherapy or of ALL itself is currently unknown. Longitudinal assessments in patients before, during, and after treatment are needed to adjudicate between these possible causes and to determine whether and how brain abnormalities evolve during and after chemotherapy. Prior longitudinal studies in childhood ALL, however, have been limited by either the absence of pretreatment baseline assessments,^1,23–25^ cognitive measures,^1,26,27^ multimodal MRI data,^1,23,24,27^ assessments in healthy controls,^1,23,24,26–28^ or data during chemotherapy.^29^

To address these limitations, we conducted a prospective, longitudinal study of 19 consecutively recruited children with newly diagnosed ALL who were treated with MTX chemotherapy, as well as 20 age- and sex-matched healthy controls. We acquired multimodal MRIs and neuropsychological assessments at a pre-methotrexate baseline within the first week of beginning treatment, and then longitudinally at weeks 9 and 22 during chemotherapy, and at 1-year follow-up, allowing us to disentangle the effects of acute illness from those of chemotherapy, characterize the within-patient evolution of brain abnormalities in temporal relation to chemotherapy administration, ascertain whether brain and cognitive abnormalities persist, and better constrain interpretation of the cellular basis of the brain abnormalities and their progression. Our a priori hypotheses, based on prior reports,^1,10,11^ were that: (1) brain abnormalities would worsen during chemotherapy, and (2) the worsening abnormalities would associate significantly with the number of intrathecal methotrexate (IT-MTX) doses.

## Methods

### Recruitment

We recruited consecutively treated children and adolescents with newly diagnosed ALL, 4–20 years old, from the Cancer and Blood Disease Institute at Children’s Hospital Los Angeles (CHLA) from April 2017 to January 2021. Healthy controls were recruited from Los Angeles. Detailed inclusion/exclusion criteria are provided in the eMethods-Supplement. Study procedures were approved by the Institutional Review Board of CHLA, written informed consent was obtained from parents (or patients if age > 18 years), and assent from youth < 18 years.

### Chemotherapy protocol

Patients were treated according to the standardized Children’s Oncology Group regimen for ALL,^30,31^ tailored to the risk of relapse: children 1–10 years old who have a white blood cell (WBC) count < 50,000/µL were classified as having “NCI standard risk”(SR) ALL; children > 10 years old or with WBC count > 50,000/µL were classified as “NCI high risk” (HR) ALL (eFig. 1).^32^ See eMethods-SI for the detailed protocol.

### Neuropsychological assessments

We assessed performance, verbal, and full-scale IQ, Processing Speed Index (PSI),^33^ current and past diagnoses of psychiatric disorders,^34^ depression severity,^35^ attention-deficit/hyperactivity disorder (ADHD) severity,^36^ and social functioning.^37^ See eMethods-Supplement for detailed instruments.

### MRI data

We collected multimodal MRI data on a 3.0 T Philips Achieva MRI scanner, which included (1) T1-weighted MRI to quantify cortical thickness and white matter volumes, (2) perfusion MRI to quantify regional cerebral blood flow (rCBF), (3) diffusion tensor imaging (DTI) to assess white matter microarchitecture using fractional anisotropy (FA) and average diffusivity coefficient (ADC) maps, and (4) MR spectroscopic imaging to construct maps of N-acetyl aspartate (NAA), a marker for the density of healthy neurons.^38^ MRI data were processed blind to the diagnosis and date of acquisition. See eMethods-Supplement for pulse sequence parameters and image processing procedures.

### Statistical analyzes

We used univariate repeated measures analyzes to test our a priori hypotheses. Because severe, acute illness can injure the brain, secondary analyzes evaluated whether patients relative to controls had abnormalities at their pre-MTX baseline. We conducted additional analyzes to assess the temporal evolution of brain abnormalities. Patients prior to pre-MTX baseline had received daily corticosteroids that could have contributed to baseline abnormalities, so we assessed whether cumulative corticosteroid dosing was associated with baseline brain measures. We also assessed whether brain measures were associated with cognitive measures and whether those associations in patients differed from those in controls. We explored whether leucovorin has neuroprotective effects, and we used structural equation modeling(SEM) to assess the causal relationships between changes in FA, NAA, cortical thickness, and rCBF values before and during chemotherapy. We controlled for false positives using a topological False Discovery Rate (FDR)^39^ procedure at an FDR = 0.05(eMethods-Supplement).

## Results

### Participant recruitment

**Fig. 1:**
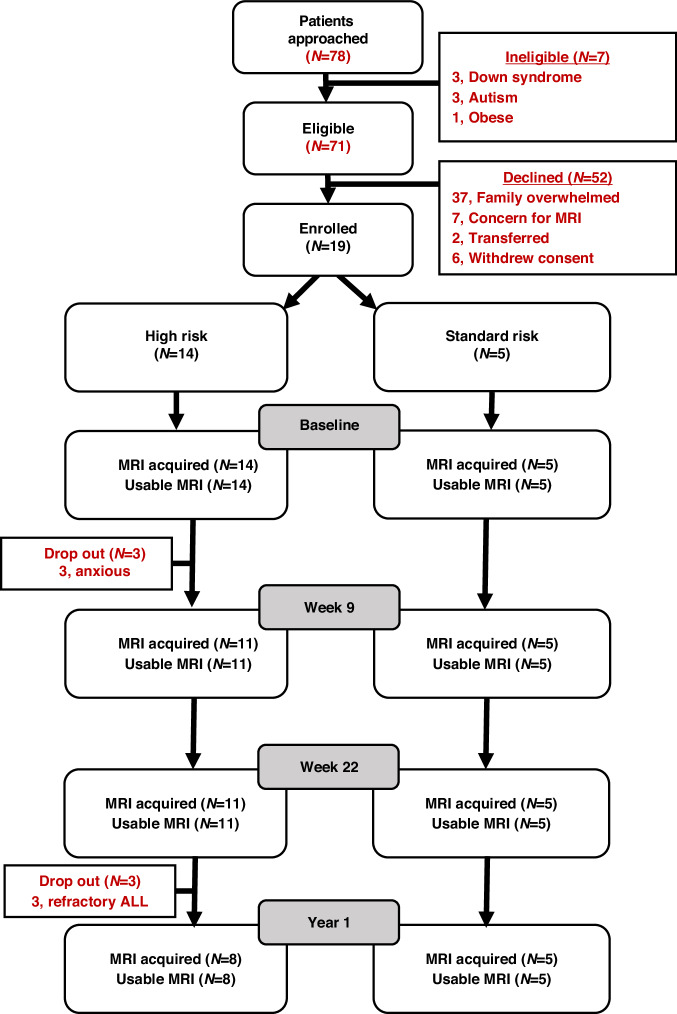
Consort diagram for patient recruitment and assessments. We approached 78 patients of whom 71 patients were eligible to participate in the study of these, 51 patients either refused to participate or withdrew consent before baseline assessments. We enrolled and collected pre-methotrexate assessments in 19 patients of whom 5 patients had SR ALL and 14 patients had HR ALL. Among the HR patients, 3 patients dropped out of the study after baseline assessments and another 3 dropped out after week 22 assessments. We collected a complete dataset at all time points in SR patients. SR standard risk, HR high risk, ALL acute lymphoblastic leukemia.

Patient recruitment and collection of pre-methotrexate baseline assessments were especially challenging because of the rapid initiation of chemotherapy within 2–3 days following ALL diagnosis (eFig. 2). We assessed 78 consecutive patients with newly diagnosed ALL, of whom 71 patients were eligible to participate in the study. Of the 71 eligible patients, 14 with HR and 5 with SR ALL consented to participate (Fig. 1). Three HR patients dropped out after baseline assessments and were not included in analyzes (eResults-Supplement). We collected baseline data in 20 controls and year 1 data in 15 controls.

### Demographics

Patients did not differ from healthy controls on sex or age at baseline or year 1 (Table 1). Out of the 19 patients, 2 patients self-identified as non-Hispanic Caucasian and 17 patients as Hispanic white. Patients relative to healthy controls were predominantly Hispanic white (*p* = 0.02, Chi-squared = 5.4, dof = 1) as 10 controls self-identified as Hispanic whites, four controls as Asians, and six controls as non-Hispanic Caucasian.

**Table 1:** Participant demographics, cognitive performance, and behavioral functioning.

		Baseline			Year 1		
		Patients (*N* = 19)	Healthy (*N* = 20)	*p*-value	Patients (*N* = 13)	Healthy (*N* = 15)	*p*-value
	Age	13.3 ± 6.3	12.9 ± 4.0	0.81 (t = 0.23,df = 37)	12.8 ± 5.2	15.0 ± 3.7	0.2 (t = 1.3,df = 26)
	Sex	16 M/3 F	18 M/2 F	0.95 $({\chi }^{2}=0.004,{df}=1)$)	13 M/0 F	14 M/1 F	0.34 $({\chi }^{2}=0.90,{df}=1)$)
Cognitive performance	FSIQ	96.4 ± 13.0	108.1 ± 12.6	**0.03** (t = −2.3, df = 21)	99.8 ± 17.6	108.0 ± 11.9	0.18 (t = −1.4, df = 18)
	VIQ	95.9 ± 9.9	105.4 ± 10.3	**0.02** (t = −2.4, df = 22)	99.5 ± 20.3	102.1 ± 9.0	0.7 (t = −0.4, df = 14)
	PIQ	98.6 ± 14.6	109.8 ± 19.0	0.09 (t = −1.75, df = 26)	101.1 ± 14.9	113.2 ± 16.0	**0.05** (t = −2.07,df = 25)
Cognitive subtests	Block design	40.6 ± 14.5	53.6 ± 15.9	**0.03** (t = −2.25, df = 25)	47.0 ± 15.2	56.6 ± 11.2	0.08 (t = −1.85,df = 19)
	Vocabulary	42.1 ± 17.2	51.8 ± 12.4	0.11 (t = −1.67, df = 19)	46.0 ± 16.6	53.7 ± 5.6	0.15 (t = −1.5, df = 13)
	Matrix reasoning	42.6 ± 15.3	52.8 ± 16.9	0.11 (t = −1.66, df = 25)	46.0 ± 11.3	58.5 ± 8.7	**0.004** (t= −3.18,df = 20)
	Similarities	39.8 ± 15.7	49.4 ± 12.8	0.1 (t = −1.72, df = 21)	46.7 ± 16.9	49.0 ± 6.9	0.7 (t = −0.43, df = 14)
	Digit span forward	9.1 ± 2.2	11.0 ± 2.8	**0.03** (t = −2.27, df = 33)	8.3 ± 2.7	10.9 ± 2.9	**0.03** (t = −2.29, df = 26)
	Digit span backward	9.8 ± 2.7	11.2 ± 3.2	0.19 (t = 1.33, df = 33)	9.1 ± 2.9	12.7 ± 3.8	**0.01** (t = −2.77, df = 26)
	Digit span total	9.6 ± 2.6	11.6 ± 2.7	**0.03** (t = −2.25, df = 31)	9.0 ± 3.8	12.0 ± 3.9	0.06 (t = −1.94, df = 26)
	Coding	8.2 ± 2.4	10.6 ± 3.6	**0.03** (t = −2.3, df = 32)	9.7 ± 2.8	10.9 ± 3.0	0.24 (t = 1.19, df = 23)
	Symbol search	8.8 ± 3.1	11.0 ± 2.9	**0.05** (t = −2.05, df = 27)	10.6 ± 3.4	12.6 ± 3.3	0.11 (t = 1.65, df = 21)
Emotional and behavioral functioning	Depression (CDRS)	26.8 ± 7.3	22.3 ± 6.8	0.18 (t = −1.4, df = 14)	20.9 ± 2.8	22.0 ± 6.6	0.67 (t = 0.44, df = 10)
	Inattention	4.1 ± 2.6	3.4 ± 4.4	0.52 (t = 0.64, df = 33)	3.6 ± 3.8	4.3 ± 4.5	0.67 (t = −0.44, df = 21)
	Hyperactivity	1.5 ± 1.6	1.8 ± 2.5	0.62 (t = −0.5, df = 34)	1.9 ± 2.8	1.1 ± 1.4	0.44 (t = 0.8, df = 12)
	ADHD	5.6 ± 3.5	5.2 ± 5.4	0.78 (t = 0.28, df = 34)	5.5 ± 5.9	5.5 ± 5.3	0.99 (t = 0.01, df = 18)
	Awareness (SRS)	49.8 ± 8.8	50.6 ± 8.0	0.83 (t = 0.33, df = 24)	48.8 ± 9.1	51.7 ± 8.3	0.52 (t = 0.66, df = 13)
	Cognition (SRS)	47.8 ± 8.4	47.8 ± 7.1	0.99(t = −0.008,df = 24)	47.9 ± 8.0	49.3 ± 7.4	073. (t = 0.36, df = 13)
	Communication (SRS)	48.1 ± 10.6	47.3 ± 7.0	0.82 (t = −0.23, df = 23)	45.4 ± 6.2	47.9 ± 4.9	0.41 (t = 0.86, df = 13)
	Motivation (SRS)	48.6 ± 10.9	49.7 ± 13.3	0.83 (t = 0.21, df = 21)	48.9 ± 9.7	49.1 ± 4.1	0.94 (t = 0.07, df = 10)
	RRB (SRS)	47.7 ± 6.4	45.6 ± 5.9	0.39 (t = −0.88, df = 24)	46.6 ± 4.6	44.7 ± 2.6	0.34 (t = −0.99, df = 11)
	Communication and interaction (SRS)	48.1 ± 9.5	48.2 ± 8.4	0.98 (t = 0.03, df = 24)	46.9 ± 8.0	49.0 ± 5.8	0.56 (t = 0.59, df = 13)
	SRS total	48.0 ± 8.5	47.5 ± 7.7	0.88 (t = −0.16, df = 24)	46.9 ± 7.1	48.0 ± 5.0	0.73 (t = 0.35, df = 13)

*IQ* intelligence quotient, *FSIQ* full-scale IQ, *VIQ* verbal IQ, *PIQ* performance IQ, *ADHD* attention-deficit/hyperactivity disorder, *SRS* social responsiveness scale, *RRB* restricted interest and repetitive behavior, *SCI* social communication and interaction, *CDRS* Columbia depression rating scale.

*P*-values considered to be statistically significant at a significance level of 0.05 are in bold font.

### Baseline cognitive performance

Patients relative to controls at baseline had (1) lower FSIQ (*p* = 0.03, t = −2.3, df = 21), (2) poorer visuospatial reasoning on the block design (*p* = 0.03, t = −2.2, df = 25), (3) poorer working memory on digit span total (*p* = 0.03, t = −2.27, df = 33), (4) slower processing speed on coding (*p* = 0.03, t = −2.3, df = 32) and symbol search (*p* = 0.05, t = −2.05, df = 27) (Table 1). Performance speed index in patients (90.61 ± 13.67) was significantly lower (*p* = 0.008, t = 2.8, df = 30) than in controls (106.05 ± 16.25).

### Year 1 cognitive performance

Patients relative to controls had (1) lower performance IQ (*p* = 0.05, t = −2.07, df = 25), (2) poorer fluid reasoning on matrix reasoning (*p* = 0.004, t = −3.18, df = 20), and (3) poorer working memory on digit span forward (*p* = 0.03, t = −2.29, df = 26) and backward (*p* = 0.01, t = −2.77, df = 26) (Table 1). Cognitive performance did not change significantly on any measure for either patients or controls over 1 year follow-up. The poorer performance of patients at year 1 but not at baseline, in the absence of significant change, is likely attributable to (a) poorer performance in patients at baseline that did not reach statistical significance, or (b) performance either increased nonsignificantly for healthy controls or decreased nonsignificantly for patients, especially on the digit span and matrix reasoning tasks. Poorer cognitive processing at year 1 in patients could be due to either illness or chemotherapy or both.

### Emotional and behavioral functioning

Patients did not differ from controls at baseline or year 1 on symptoms of depression (*p* = 0.18, t = 1.43, df = 14), inattention (*p* = 0.53, t = 0.64, df = 33), hyperactivity (*p* = 0.62, t = −0.5, df = 34), or total ADHD symptoms (*p* = 0.78, t = 0.28, df = 34), or on social responsiveness scale (*p* = 0.88, t = −0.16, df = 24) (Table 1).

### Group differences in MRI measures

Clinical reading of T2-weighted images by an expert neurologist revealed no visually detectable lesions in the brain for any participant at any time point in the longitudinally collected data.

#### Cortical thickness

**Fig. 2:**
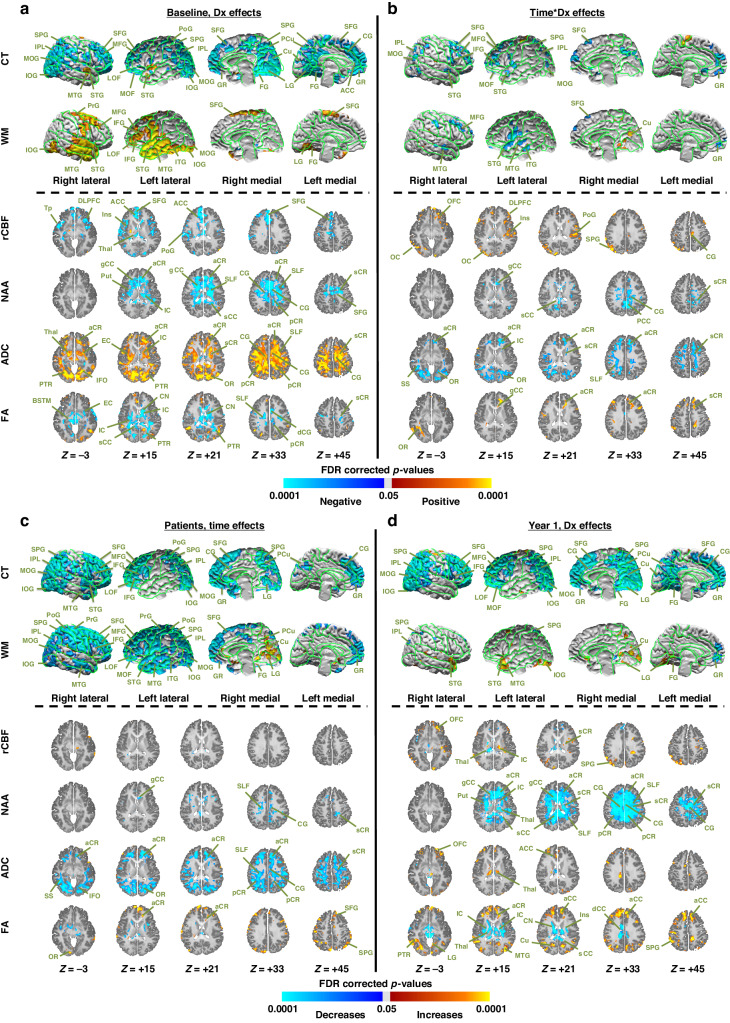
Baseline abnormalities and their progression in patients relative to healthy controls. We assessed **a** whether at pre-methotrexate baseline brain measures in patients differed from those in healthy controls (Dx Effects), **b** whether brain measures changed differentially in patients relative to healthy controls (Time × Dx effects), **c** how brain measures changed within patients alone (Time effects), and **d** brain abnormalities at year 1 in patients relative to healthy controls. We controlled for nuisance effects of age and computed the statistical significance of the diagnosis effects on the brain. We controlled for false positives in multiple hypotheses testing using a procedure for Topological FDR; *P*-values that survived this procedure were color encoded and displayed either on the surface (rows *CT and WM*) or on the axial slices (rows *rCBF, NAA, ADC, FA*) of the template brain. Violet and blue show significantly lower values whereas yellow and red show significantly higher values in patients relative to controls. Dx diagnosis, CT cortical thickness, WM white matter, rCBF regional cerebral blood flow, NAA N-acetyl aspartate, ADC average diffusivity coefficient, FA fractional anisotropy, SFG superior frontal gyrus, MFG middle frontal gyrus, IFG inferior frontal gyrus, DLPFC dorsolateral prefrontal cortex, LOF lateral orbitofrontal gyrus, MOF middle orbitofrontal gyrus, SPG superior parietal gyrus, MPG middle parietal gyrus, STG superior temporal gyrus, MTG middle temporal gyrus, ITG inferior temporal gyrus, MOG middle occipital gyrus, IOG inferior occipital gyrus, PoG postcentral gyrus, PrG precentral gyrus, CG cingulate gyrus, dCG dorsal cingulate gyrus, ACC anterior cingulate cortex, PCC posterior cingulate cortex, Tp temporal pole, Cu cuneus, PCu precuneus, LG lingual gyrus, GR gyrus rectus, FG fusiform gyrus, CN caudate nucleus, Put putamen, Thal thalamus, SLF superior longitudinal fasciculus, gCC genu of corpus callosum, sCC splenium of corpus callosum, EC external capsule, IC internal capsule, aCR anterior corona radiata, sCR superior corona radiata, pCR posterior corona radiata, PTR posterior thalamic radiations, OR optic radiations, PTR posterior thalamic radiations, IFO inferior frontal-occipital fasciculus.

At their pre-MTX baseline, patients relative to controls had significantly thinner cortices bilaterally across most of the cortical surface, especially in lateral and mesial portions of frontal, parietal, and occipital lobes (Fig. 2a, eFigs. 8, 14A). Cortical thickness over the first year of treatment changed significantly in patients compared to controls, as evidenced by the time*group interaction (Fig. 2b, eFigs. 9, 14B), deriving from significantly more cortical thinning over time in patient frontal, parietal, and anterior temporal cortices (Fig. 2c, eFig. 15C), yielding thinner cortices in these regions for patients at Year 1 (Fig. 2d, eFig. 15D).

#### White matter surface

Patients compared with controls had larger WM volumes bilaterally in the parietal and temporal lobes, dorsolateral prefrontal cortex, and mesial superior frontal gyrus (Fig. 2a, eFig. 8, 14A). WM volumes changed significantly in patients compared to controls as evidenced by the time*group interaction (Fig. 2b, eFig. 9, 14B), which derived from significant declines in WM volumes across most of the lateral brain surface in patients (Fig. 2c, eFig. 15C). Consequently, by year 1, WM volumes in patients did not differ from healthy controls except for larger volumes in the temporal pole bilaterally and right cuneus, and smaller volumes in the left gyrus rectus (Fig. 2d, eFig. 15D).

#### rCBF

Patients compared with controls at baseline had significantly lower rCBF in gray matter of the prefrontal, temporal, and parietal lobes and thalamus (Fig. 2a, eFig. 8, 14A). Over the 1 year of treatment, rCBF changed differentially in patients relative to controls (Fig. 2b, eFig. 9, 14B), with rCBF declining in controls (eFig. 7) but not in patients (Fig. 2c, eFig. 15C), leading to significantly higher rCBF in cortical gray matter but lower in thalamus in patients at 1 year.

#### NAA

Patients relative to controls at baseline had significantly lower NAA levels in WM throughout anterior brain regions (Fig. 2a and eFigs. 8, 14A). NAA declined significantly more in patients than controls in WM and posterior cingulate gyrus (Fig. 2b, c, eFigs. 8, 9, 14), leading to significantly lower NAA across larger expanses of WM and cingulate cortex in patients at 1 year (Fig. 2d, eFig. 15D).

#### DTI: ADC & FA

Patients at baseline had significantly higher ADC but lower FA values throughout large portions of WM (Fig. 2a, eFig. 8, 14A). Over the year of treatment, changes in these values differed significantly across groups (Fig. 2b, eFig. 9, 14B), largely from decreases in ADC in WM and increases in FA in cortical gray matter (Fig. 2c and eFig. 15C) in patients without changing in controls (eFig. 6). Consequently, at year 1, patients had significantly higher ADCs in cortical gray matter and thalamus and higher FA in WM, but lower FA in subcortical gray matter.

### Brain associations with IT-MTX dosing

#### Cortical thickness

**Fig. 3:**
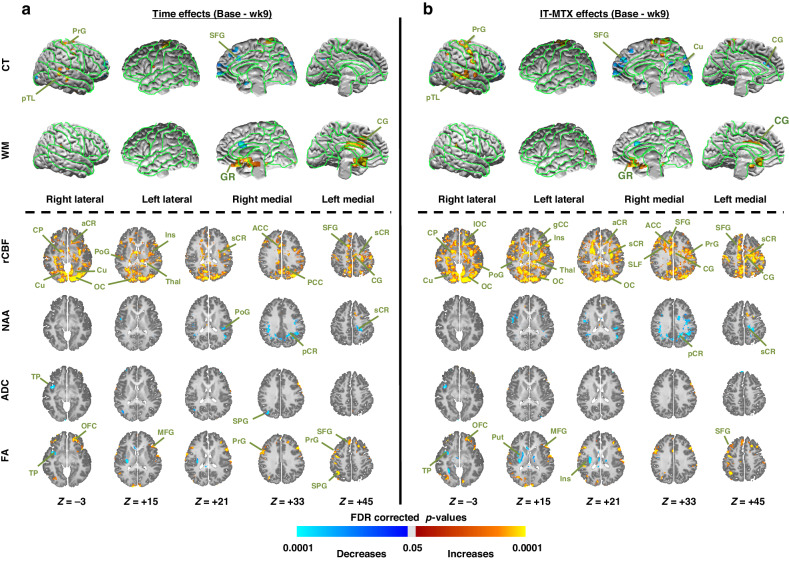
Baseline to week 9 changes in brain measures (left panel) and their associations with IT-MTX dose (right panel) within the patient group. We conducted univariate repeated measures analyzes using baseline and week 9 data within patients alone to assess separately (**a**) how MRI-derived brain measures changed from baseline to week 9 (left panel, Time effects); and (**b**) how those changes were associated with IT-MTX dose (right panel, IT-MTX effects). We controlled for nuisance effects of age and risk of the time or the IT-MTX dose effects on the brain. We controlled for false positives in multiple hypotheses testing using a procedure for Topological FDR; P-values that survived this procedure were color encoded and displayed either on the surface (rows *CT and WM*) or on the axial slices (rows *rCBF, NAA, ADC, FA*) of the template brain. Violet and blue show significant decreases whereas yellow and red show significant increases in patients from baseline to week 9. CT cortical thickness, WM white matter, rCBF regional cerebral blood flow, NAA N-acetyl aspartate, ADC average diffusivity coefficient, FA fractional anisotropy, SFG superior frontal gyrus, MFG middle frontal gyrus, IFG inferior frontal gyrus, DLPFC dorsolateral prefrontal cortex, LOF lateral orbitofrontal gyrus, MOF middle orbitofrontal gyrus, SPG superior parietal gyrus, MPG middle parietal gyrus, STG superior temporal gyrus, MTG middle temporal gyrus, ITG inferior temporal gyrus, MOG middle occipital gyrus, IOG inferior occipital gyrus, PoG postcentral gyrus, PrG precentral gyrus, CG cingulate gyrus, dCG dorsal cingulate gyrus, ACC anterior cingulate cortex, PCC posterior cingulate cortex, Tp temporal pole, Cu cuneus, PCu precuneus LG lingual gyrus, GR gyrus rectus, FG fusiform gyrus, CN caudate nucleus, Put putamen, Thal thalamus, SLF superior longitudinal fasciculus, gCC genu of corpus callosum, sCC splenium of corpus callosum, EC external capsule, IC internal capsule, aCR anterior corona radiata, sCR superior corona radiata, pCR posterior corona radiata, PTR posterior thalamic radiations, OR optic radiations, PTR posterior thalamic radiations, IFO inferior frontal-occipital fasciculus.

**Fig. 4:**
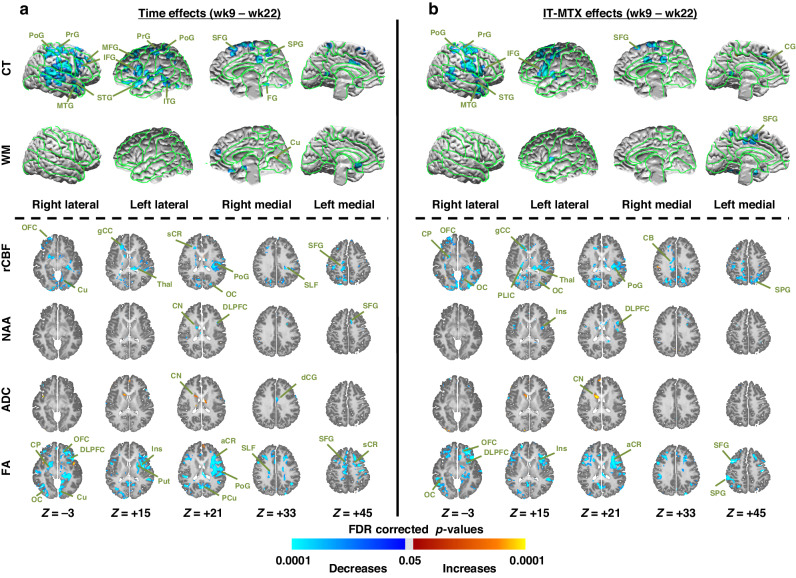
Week 9 to week 22 changes in brain measures (left panel) and their associations with IT-MTX Dose (right panel) within the patient group. We conducted univariate repeated measures analyzes using week 9 and week 22 data within patients alone to assess separately (**a**) how MRI-derived brain measures changed from week 9 to week 22 (left panel, Time effects); and (**b**) how those changes were associated with IT-MTX dose (right panel, IT-MTX effects). We controlled for nuisance effects of age and risk of the time or the IT-MTX dose effects on the brain. We controlled for false positives in multiple hypotheses testing using a procedure for Topological FDR; *P*-values that survived this procedure were color encoded and displayed either on the surface (rows *CT and WM*) or on the axial slices (rows *rCBF, NAA, ADC, FA*) of the template brain. Violet and blue show significant decreases whereas yellow and red show significant increases in patients from baseline to week 9. CT cortical thickness, WM white matter, rCBF regional cerebral blood flow, NAA N-acetyl aspartate, ADC average diffusivity coefficient, FA fractional anisotropy, SFG superior frontal gyrus, MFG middle frontal gyrus, IFG inferior frontal gyrus, DLPFC dorsolateral prefrontal cortex, LOF lateral orbitofrontal gyrus, MOF middle orbitofrontal gyrus, SPG superior parietal gyrus, MPG middle parietal gyrus, STG superior temporal gyrus, MTG middle temporal gyrus, ITG inferior temporal gyrus, MOG middle occipital gyrus, IOG inferior occipital gyrus, PoG postcentral gyrus, PrG precentral gyrus, CG cingulate gyrus, dCG dorsal cingulate gyrus, ACC anterior cingulate cortex, PCC posterior cingulate cortex, Tp temporal pole, Cu cuneus, PCu precuneus LG lingual gyrus, GR gyrus rectus, FG fusiform gyrus, CN caudate nucleus, Put putamen, Thal thalamus, SLF superior longitudinal fasciculus, gCC genu of corpus callosum, sCC splenium of corpus callosum, EC external capsule, IC internal capsule, aCR anterior corona radiata, sCR superior corona radiata, pCR posterior corona radiata, PTR posterior thalamic radiations, OR optic radiations, PTR posterior thalamic radiations, IFO inferior frontal-occipital fasciculus.

**Fig. 5:**
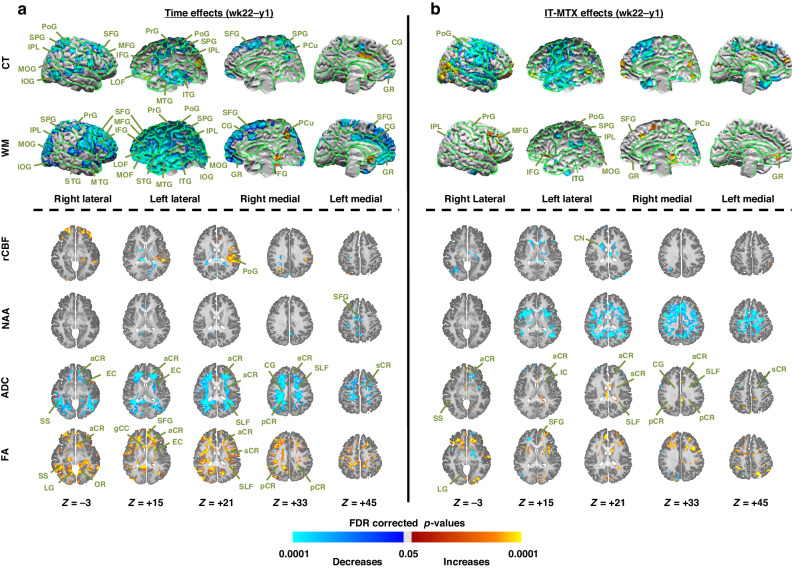
Week 22 to Year 1 changes in brain measures (left panel) and their associations with IT-MTX dose (right panel) within the patient group. We conducted univariate repeated measures analyzes using week 22 and year 1 data within patients alone to assess separately (**a**) how MRI-derived brain measures changed from week 22 to year 1 (left panel, Time effects); and (**b**) how those changes were associated with IT-MTX dose (right panel, IT-MTX effects). We controlled for nuisance effects of age and risk of the time or the IT-MTX dose effects on the brain. We controlled for false positives in multiple hypotheses testing using a procedure for Topological FDR; P-values that survived this procedure were color encoded and displayed either on the surface (rows *CT and WM*) or on the axial slices (rows *rCBF, NAA, ADC, FA*) of the template brain. Violet and blue show significant decreases whereas yellow and red show significant increases in patients from baseline to week 9. CT cortical thickness, WM white matter, rCBF regional cerebral blood flow, NAA N-acetyl aspartate, ADC average diffusivity coefficient, FA fractional anisotropy, SFG superior frontal gyrus, MFG middle frontal gyrus, IFG inferior frontal gyrus, DLPFC dorsolateral prefrontal cortex, LOF lateral orbitofrontal gyrus, MOF middle orbitofrontal gyrus, SPG superior parietal gyrus, MPG middle parietal gyrus, STG superior temporal gyrus, MTG middle temporal gyrus, ITG inferior temporal gyrus, MOG middle occipital gyrus, IOG inferior occipital gyrus, PoG postcentral gyrus, PrG precentral gyrus, CG cingulate gyrus, dCG dorsal cingulate gyrus, ACC anterior cingulate cortex, PCC posterior cingulate cortex, Tp temporal pole, Cu cuneus, PCu precuneus LG lingual gyrus, GR gyrus rectus, FG fusiform gyrus, CN caudate nucleus, Put putamen, Thal thalamus, SLF superior longitudinal fasciculus, gCC genu of corpus callosum, sCC splenium of corpus callosum, EC external capsule, IC internal capsule, aCR anterior corona radiata, sCR superior corona radiata, pCR posterior corona radiata, PTR posterior thalamic radiations, OR optic radiations, PTR posterior thalamic radiations, IFO inferior frontal-occipital fasciculus.

Cortical thickness did not change significantly from baseline to week 9 (Fig. 3a), but from week 9 to 22 it thinned significantly in the temporal and sensorimotor cortices (Fig. 4a) in association with IT-MTX dosing (Fig. 4b). The cortex continued to thin significantly in these regions from week 22 to year 1 (Fig. 5a), with higher doses of IT-MTX associated with more thinning of temporal, parietal, and inferior frontal cortices (Fig. 3b).

#### White matter surface

Local WM volumes did not change significantly from baseline to week 9 (Fig. 3a) or from week 9 to 22 (Fig. 4a), but they declined significantly from week 22 to year 1 (Fig. 5a), with declines sparsely associated with IT-MTX (Fig. 5b).

#### rCBF

increased from baseline to week 9 (Fig. 3a) and then decreased from week 9 to 22 (Fig. 4a) in cortical gray matter, both in direct proportion to IT-MTX dosing (Figs. 3b, 4b). rCBF then increased in cortical gray matter but declined in white matter from week 22 to year 1 (Fig. 5a), unassociated with IT-MTX dosing.

#### DTI

ADC did not change from baseline to week 9 (Fig. 3a) or from week 9 to 22 (Fig. 4a), then declined significantly in WM from week 22 to year 1 (Fig. 5a), unassociated with IT-MTX dosing (Fig. 5b). FA increased in cortical gray matter from baseline to week 9 (Fig. 3a), then declined from week 9 to 22 (Fig. 4b) in proportions to IT-MTX dosing (Figs. 3b, 4b). From week 22 to year 1, FA increased significantly in WM (Fig. 5a), unassociated with IT-MTX dose (Fig. 5b). AD and RD did not change from baseline to week 9 (eFig. 11A) or from week 9 to 22 (eFig. 12A), then declined significantly in WM from week 22 to year 1 (eFig. 13A), in direct proportions with IT-MTX dosing (eFig. 13B). Q-space imaging (QSI) measures of isotropic diffusion (ISO) and non-restricted diffusion imaging (NRDI) for edema increased slightly from baseline to week 9 (eFig. 11A) in direct proportion to IT-MTX dosing (eFig. 11B). ISO and NRDI measures for edema and restricted diffusion imaging (RDI) for cellular infiltration subsequently decreased from week 9 to week 22 largely in GM in direct proportions to IT-MTX dosing (eFig. 12); and did not change from week 22 to year 1, except for increases in the thalamus (eFig. 13).

#### Steroid and leucovorin dosing

More steroid doses were significantly associated with thicker cortex, higher rCBF, and higher NAA in superior brain regions, and lower FA in gray matter (eFigs. 3, 10). Associations of performance speed with brain measures in patients did not differ significantly from those in controls (eFig. 4). Higher leucovorin doses were associated with thicker cortices, larger WM volumes, higher NAA levels, greater ADC in gray matter, higher FA in WM, and lower rCBF in gray matter but higher rCBF in WM (eFig. 5).

#### SEM analyzes

showed that baseline associations of NAA with cortical thickness were disrupted at week 9 and week 22 but were restored by year 1 (eFig. 7).

## Discussion

Patients relative to controls had cognitive deficits at a pre-methotrexate baseline, within the first week of treatment (eFig. 2), that persisted to year 1 of chemotherapy. Patients did not have significantly more behavioral or emotional problems at any time point. They also had widespread abnormalities in brain structure, tissue microstructure, rCBF, and metabolite concentrations at baseline (Fig. 2). Some of the baseline abnormalities worsened, some remained stable, and some normalized over 1-year follow-up.

Cognitive deficits and brain abnormalities at baseline could derive from either the very rapid effects of the single dose of intrathecal cytarabine, the initial administration of corticosteroids, or the acute illness itself and its associated effects on systemic physiology, metabolism, inflammation, and oxidative stress. Cumulative corticosteroid dosages, however, were unassociated with baseline WM abnormalities (eFig. 3), and higher cumulative doses were associated with thicker cortices, lower FA values, and higher cortical rCBF (eFig. 3)—all generally opposite the direction of baseline abnormalities, suggesting that steroids were neuroprotective, consistent with prior research that they help to reduce inflammation,^40^ restore the integrity and function of blood–brain barrier,^41–43^ and reduce brain swelling.^40^

Patients had thinner cortices at baseline that worsened progressively over time after week 9 in direct proportion to IT-MTX dosing. Leucovorin rescue partially countered these effects from week 9 to 22 (eFig. 5). Frontal and temporal WM was enlarged at baseline but resolved over time, especially from week 22 to year 1. Thinner cortex at baseline suggests the presence of reduced dendritic arborization and synaptic density, since these cellular features are likely the primary determinants of cortical thickness measures.^44^ The reduction in dendrites and synapses may be a consequence of stress-related increases in endogenous cortisol,^45^ initial treatment with dexamethasone, increased oxidative stress associated with leukemia,^46^ or initial MTX treatment.^47^ These same factors may have contributed to progressive cortical thinning over the year, though our findings suggest that steroid treatment tended to attenuate cortical thinning (eFig. 3), and oxidative stress presumably declined over time with clinical improvement, leaving MTX as the most likely cause of continued cortical thinning over the year of treatment. The countering of thinning in proportion to leucovorin dosing during rescue from week 9 to 22 further supports the probability that at least a portion of cortical thinning is attributable to the effects of MTX.

rCBF was lower at baseline in medial prefrontal cortices, insula, and sensorimotor cortices, perhaps from lower metabolic demand as a consequence of reduced dendritic arborization and synaptic density in the cortical mantle (Fig. 2). rCBF increased from baseline to week 9 and declined steadily thereafter, leaving rCBF unchanged from baseline by year 1 (Fig. 3c). These latter rCBF changes were significantly associated with IT-MTX dosing (Figs. 4, 5), suggesting that normalization of rCBF after week 9 was a consequence of MTX-induced normalization of the effects of inflammation and oxidative stress from ALL on brain metabolism and blood flow,^48–51^ as well as perhaps the postulated effects of MTX on reducing the density of cortical dendrites and synapses, which are metabolically demanding.

The combination at baseline of enlarged WM volumes and widespread greater WM water diffusivity(ADC) suggests an increase in free water within WM, likely a microscopic extracellular exudate in response to ALL-associated inflammation. Both WM volumes and ADC normalized over the year, and with a similar time course, lending support to the possibility that the two findings represent the same underlying microscopic and cellular WM processes. Microstructural tissue organization, as indexed by FA, was reduced at baseline in posteroinferior WM, internal capsule, and caudate nucleus and it persisted throughout the year. FA also increased slightly throughout the year in frontal gray and white matter, especially from week 22 to year 1, in proportion to IT-MTX dosing. Decreases in AD and RD values from week 22 to year 1 were consistent with decreases in ADC values (eFigs. 13, 5a). However, AD and RD decreased in direct proportion to the IT-MTX dosing, thereby suggesting that decreased diffusion was a consequence of chemotherapy. In addition or alternatively, the combination of decreased ADC in white matter with increased FA (the latter in more focal and scattered locations) could suggest the presence of reduced or impaired WM myelination at baseline that normalized throughout the year, especially in posteroinferior and superior frontal WM. Normalization of myelination likely was preceded by decreases in GM and WM edema from week 9 to week 22 as suggested by decreases in ISO and NRDI, as well as decreases in cellular infiltration as suggested by the decreases in RDI (eFig. 12). White matter normalization suggests that these abnormalities were reversed by chemotherapy either directly or indirectly, via reduction of systemic inflammation or oxidative stress.

Neuronal density, as indexed by NAA levels, was reduced at baseline throughout most of anterior brain WM and in the gray matter of the basal ganglia and cingulate gyrus, thereafter remaining low or declining slightly throughout the year. These enduring abnormalities are likely a cytotoxic consequence of MTX^52^ to post-mitotic oligodendrocytes.^53^ and neurons since MTX damages DNA,^52,54,55^ reduces dendritic branching and spine density,^56^ and induces apoptosis in astrocytes.^57,58^ Cell death and suppression of cell division by chemotherapeutic drugs.^53^ also likely contribute to enduring neurological and cognitive deficits.^59^ in ALL survivors. SEM analyzes suggest that our findings across multiple MRI modalities are likely determined by treatment-associated reductions in neuronal density and inflammation-induced cerebral edema (eFig. 7).

### Systemic and CNS Inflammation

ALL is associated with elevated pro- and anti-inflammatory cytokines and chemokines.^60^ Although the brain is isolated from peripheral immune cells and cytokines,^61^ ALL produces systemic hyperactivation and proliferation of immune cells that increase the permeability of the blood–brain barrier,^62,63^ astrocytic aquaporin-4 expression, and water permeability, which in turn cause cerebral edema.^64^ Consequently, cytokines passively diffuse from the systemic circulation into the brain,^65^ activating microglia and astrocytes.^66,67^ Animal models have shown that MTX exposure activates microglia,^68^ which then proliferate and release pro-inflammatory cytokines that are cytotoxic to oligodendrocytes and their precursors,^69,70^ inhibit oligodendrocyte differentiation, reduce mature oligodendrocytes,^71^ release cytotoxic reactive oxygen species including hydrogen peroxide and nitric oxide,^72,73^ secrete glutamate, aspartate, and quinolinic acid that can be neurotoxic,^74^ and prune synapses and dendritic branches.^75,76^ Neuroinflammation could have been exacerbated by intrathecal cytarabine, which has been associated with inflammation in and damage to myelin.^77^

Neuroinflammation caused by either acute illness or cytarabine administration could therefore account for all findings detected at our pretreatment baseline, within the first week of chemotherapy. The inflammation-induced increase in water permeability could account for cerebral edema and subtle brain swelling. Injury to oligodendrocytes and their precursor cells could account for diffuse white matter injury. Furthermore, reactive oxygen species can damage or kill neurons, which may account for the enduring reduction in the density of healthy neurons we detected. Loss of synapses and reduced dendritic arborization likely account for the progressive cortical thinning from baseline through the first year of treatment.

### Limitations

Our findings should be viewed in light of several limitations. First, chemotherapy was initiated within 2–3 days of diagnosis (eFig. 2), limiting the time window for baseline assessments. The slight delay in baseline assessments confounds the effects of acute illness with those of corticosteroids and single-dose intrathecal cytarabine administered before our assessments. Second, our sample size was small because of the rapid initiation of chemotherapy, thereby limiting the number of patients we could approach, consent, and collect baseline assessments prior to methotrexate treatment. Although the small sample size decreased our statistical power to detect small effect sizes, our longitudinal study design showed the presence of medium-to-large effects of illness at baseline and progressive effects of IT-MTX dose, steroid dose, leucovorin rescue differentially on GM and WM of the brain in the underserved, LatinX community. Third, because patients were predominantly males, we could not assess the moderating effects of sex on our findings. Fourth, our participants were predominantly Hispanic, thereby limiting the generalizability of the findings. Patients comprised significantly more Hispanic whites than healthy controls, which possibly could have confounded the effects of diagnosis on baseline and year 1 findings. Fifth, the risk of relapse was confounded with both age and the differing chemotherapy protocols: SR patients received “Capizzi” MTX, whereas HR patients received high-dose MTX, during interim maintenance. Confounding of risk with age and chemotherapy protocol precluded assessment of whether HD-MTX is more neurotoxic than “Capizzi” MTX.

### Conclusions

Our findings suggest that brain abnormalities at pre-MTX baseline are likely attributable to the oxidative stress and inflammation associated with acute illness. Some of these abnormalities, particularly those in WM that we regard as inflammation-induced cerebral edema, resolved over the first year of treatment. Steroid treatment attenuated these abnormalities prior to MTX administration, likely by reducing ALL-induced neuroinflammation. MTX was most likely responsible for the exacerbation of cortical thinning observed over the year of treatment. Our findings suggest that the administration of neuroprotective and anti-inflammatory agents, including corticosteroids, should be considered even earlier than currently administered after the diagnosis of ALL. Evidence for the neuroprotective effects of leucovorin suggests that strategies may be developed to extend the duration of this intervention or adapt it for use in SR patients.

## Supplementary Information

SUPPLEMENTARY MATERIAL

## Data Availability

Deidentified MRI and neuropsychological data will be freely available to for research purposes upon written requests.
