# Supplementary material for: Progression of brain injuries associated with methotrexate chemotherapy in childhood acute lymphoblastic leukemia
Source: Pediatr Res. 2024 Jul 1;97(1):348–59. doi: 10.1038/s41390-024-03351-9 (PMC11798858; doi:10.1038/s41390-024-03351-9)
Supplement: Supplementary file 1 — SUPPLEMENTARY MATERIAL [file 41390_2024_3351_MOESM1_ESM.pdf]

SUPPLEMENTARY MATERIAL

Table of Contents

eMETHODS 3
Inclusion/Exclusion Criteria 3
Chemotherapy Protocol 3
Neuropsychological Assessments 3
MRI Data Acquisition 4
MRI Data Processing 4
Statistical Analyses 7
eRESULTS 10
Participant Recruitment and Retention 10
Baseline Assessments 10
Corticosteroid Associations with Brain Measures 11
Performance Speed Associations with Brain Measures 11
Leucovorin Rescue 11
Baseline to Year 1 Changes in Brain Measures in Healthy Controls 11
Cross-Sectional Relationships among Multimodal Brain Measures 11
eDISCUSSION 12
Relationship to Prior Studies 12
Brain Networks Subserving Attention and Working Memory 13
MTX Toxicity to Mature, Post-Mitotic Cells 14
Structural Equation Modeling 14
eREFERENCES 15
eFIGURES 20
eFigure 1: Study Assessments and Children's Oncology Group Chemotherapy Treatment Protocol 20
eFigure 2: Distribution of Days 22
eFigure 3: Associations of Cumulative Corticosteroid Dose with Baseline Brain Measures 23
eFigure 4 A&B: Associations of Performance Speed Index (PSI) with Baseline Brain Measures 24
eFigure 4 C: Associations of Performance Speed Index (PSI) with Baseline Brain Measures 25
eFigure 5: Associations of Leucovorin Rescue with Changes in Brain 26
eFigure 6: Changes in MRI-Derived Brain Measures within Healthy Controls 28
eFigure 7: Structural Equation Model (SEM) for Causal Relation among MRI-Derive Measures 30
eFigure 8: Bar Graphs for Diagnosis Effects at Pre-Methotrexate Baseline 31
eFigure 9: Bar Graphs for Differing Time Effects 32
eFigure 10: Scatterplots for Associations of Cumulative Steroid Dose 33
eFigure 11: Baseline to Week 9 changes in Multi-Shell DTI and QSI Brain Measures 34
eFigure 12: Week 9 to Week 22 changes in Multi-Shell DTI and QSI Brain Measures 35

eFigure 13: Week 22 to Year 1 changes in Multi-Shell DTI and QSI Brain Measures 37
eFigure 14: Baseline Abnormalities and their Differential Progression in Patients relative to Healthy Controls 38
eFigure 15: Progression and Persistence of Abnormalities in Patients 39

eMETHODS

Inclusion/Exclusion Criteria All patients, ages 4 to 20 years, newly diagnosed with ALL who were scheduled to undergo conventional methotrexate (MTX) chemotherapy. Patients anticipated to receive cranial radiation therapy, as it is known to produce widespread brain and cognitive disturbances that differ from those of MTX. Exclusion criteria for controls included any lifetime history of psychiatric disorder. Additional exclusion criteria for both groups include a diagnosis of prior neurological illness or Autism Spectrum Disorder, Down syndrome, an IQ less than 70, a serious or uncontrolled chronic medical illness (other than ALL), or contraindication to MRI scanning.

Chemotherapy Protocol Patients were treated according to the standardized Children's Oncology Group regimens for ALL (**eFig.1**):[1,2] (**1**) **Induction**: At the beginning of induction, patients received a single dose of intrathecal cytarabine (an inhibitor of DNA synthesis), then 2-4 doses of intrathecal MTX (IT-MTX), depending on age and CNS disease burden, over 4 weeks. HR patients also received 60 mg/m^2 of prednisone daily, and SR patients 6 mg/m^2 of dexamethasone daily, for those 4 weeks. **(2) Consolidation**: SR patients received 3 doses of IT-MTX over 4 weeks, and HR patients received 4 doses of IT-MTX over 8 weeks; **(3) Interim maintenance #1** is when treatment diverged for HR and SR patients. SR patients received escalating doses of intravenous MTX ("Capizzi"), dose range 100-300 mg/m^2, and 1 dose of IT-MTX. HR patients received 4 courses of high dose (5000 mg/m^2) MTX intravenously over 24 hours and, simultaneously, 4 courses of leucovorin beginning 42 hours after the start of MTX infusion. Leucovorin, an active metabolite of folic acid and an essential coenzyme for nucleic acid synthesis, was used to selectively "rescue" non-leukemia cells from the adverse effects of high dose MTX and thereby reduce systemic toxicity.[3] The dose and duration of leucovorin during rescue was adjusted based on the clearance and toxicity observed in prior courses. HR patients also received 2 doses of IT-MTX. **(4) Delayed intensification**: SR patients received 2 doses, and HR patients received 3 doses, of intrathecal MTX over 8 weeks. All patients received 10 mg/m^2 of dexamethasone daily for 14 days. **(5) Interim maintenance #2**: All patients, except for 2 with HR ALL, received Capizzi MTX (C-MTX), 2 doses of intrathecal MTX, and 10 mg/m^2 dexamethasone daily for 14 days. (**6) 12-week maintenance:** all patients were treated with 1-2 doses of IT-MTX in each 3-month cycle and 40 mg/m^2 of prednisone daily for SR ALL or 6 mg/m^2 dexamethasone daily HR ALL.

Neuropsychological Assessments

We administered the Block Design, Matrix Reasoning, Vocabulary, and Similarities subtests from the Wechsler Preschool and Primary Scale of Intelligence-IV (WPPSI-IV)[4] in children younger than 6 years of age or from the Wechsler Abbreviated Scale of Intelligence (WASI-II)[5] in youth 6 years or older. We used age-standardized T-scores for Block Design and Matrix Reasoning to compute Performance IQ and T-scores for Vocabulary and Similarities to compute Verbal IQ. All 4 subtests were used to compute FSIQ. We also administered the Digit Span, Digit-Symbol Coding, and Symbol Search subtests from the Wechsler Intelligence Scale for Children-5th Edition (WISC-V)[6] in youth >6 years but <17 years or from the Wechsler Adult Intelligence Scale, Fourth Edition (WAIS-IV)[7] in participants >17 years. We combined the scaled scores for Coding and Symbol Search subtests to compute a Processing Speed Index (**PSI**).[7]

We used the Kiddie Schedule for Affective Disorders and Schizophrenia, Present and Lifetime Version (KSADS-PL-5)[8] to assess current and past diagnoses of major psychiatric disorders in all participants. We assessed emotional and behavioral functioning using (a) Children's Depression Rating Scale, Revised (CDRS-R) for depression severity;[9] (b) parental and teacher reports for attention-deficit/hyperactivity (ADHD) symptoms;[10] and (c) social awareness, cognition, awareness, motivation, repetitive behavior, communication, and interaction using Social Responsiveness Scale 2.[11]

MRI Data Acquisition MRI data were acquired on a 3.0T Philips Achieva MRI scanner equipped with a 32-channel, phased array head coil. Participants were familiarized with scanning procedures in a mock scanner on the morning of a scan. All MR data were acquired without the use of contrast agents or sedation.

T1-Weighted MRI We acquired high resolution T1- weighted anatomical MRI using a 3D gradient echo pulse sequence with repetition time (TR) = 8.9 ms, echo time (TE) = 3.4 ms, flip angle (FA) = 8°, slice thickness = 1mm, number of slices=170, field of view (FOV) = 25 cm, Phase Field of View (pFOV)=100%; matrix size = 256 x 256, turbo spin echo (TSE) factor = 128, and number of excitations (NEX) = 1. The T1-weighted sequence was repeated to acquire two images of NEX = 1, and were then coregistered and averaged during post processing.

Diffusion Tensor Imaging (DTI) We acquired multi-shell, high angular resolution DTI data with TR=5,300 ms, TE=89 ms, FA=78°, matrix size 128 x 128, FOV=24 cm, number of slices = 72, slice thickness = 2 mm, b = 500, 1000, 2000, and 3000 s/mm^2, diffusion gradient directions = 102, 3 B0 images, and echo planar imaging (EPI) factor = 119.

Perfusion MRI We acquired perfusion MRI data using 2D pseudo-continuous arterial spin labeling (2D pCASL) pulse sequence with TR=4,000 ms, TE=14 ms, FA=90°, number of slices = 28, slice thickness = 5 mm, matrix size = 96 x 96; FOV = 24 cm, post labeling delay (PLD) = 1,525 ms, 2 background suppression pulses, number of volumes = 120. We also acquired M-zero image for quantification and high-inplane resolution overlay image for normalization with the same participant's anatomical MRI in the same slices and orientation as the 2D pCASL data. The overlay image was acquired with TR=2,148 ms, TE=9.94 ms, inversion recovery (IR) = 840 ms, TSE factor = 9, FOV=24 cm, matrix size = 240 x 240, slice thickness = 5 mm, and number of slices = 28. An M-zero image was acquired with TR = 5,000 ms, TE = 9 ms, TSE factor = 26, matrix size = 88 x 86, FA=90°, number of slices = 28, slice thickness = 5 mm, and FOV=24 cm.

MR Spectroscopy The MR spectroscopy data were acquired using a multiplanar, spin echo sequence along 6 transverse slices, slice thickness = 10 mm, slice spacing = 2 mm, with 6 outer volume suppression bands, water was suppressed using a chemical shift selective suppression (CHESS) method, TR=2,300 ms, TE=144 ms, FA=90°, number of samples 512, matrix size = 24 x 24, and FOV=24 cm. We also acquired T1-w localizer image in the same slices and orientation but at higher in-plane resolution for normalization of the MRS data into the coordinate space of the same participant anatomical MRI. The localizer image was acquired a spin echo sequence with parameters IR = 840 ms, number of slices = 6, FOV=24 cm, matrix size = 240 x 240, slice thickness = 10 mm, slice spacing = 2 mm, TE= 9.94 ms, TR=2,148 ms, and TSE factor = 5.

MRI Data Processing

We processed MRI data blind to the participant diagnosis and temporal order of acquisition. Data were visually assessed for motion and zipper artifacts and signal loss. MRS data were assessed for excessive noise, baseline distortion of the spectrum, signal contamination from the scalp lipids, broadening and overlapping of choline and creatine linewidth, and incorrect placement of the outer volume suppression bands. Diffusion-weighted images were assessed for motion and systematic shift in principal directions. Data with artifacts were excluded and not processed further.

Selecting Template Brain We selected as a template the brain of a single healthy participant. The use of a single brain as the template provided sharp tissue contrast and therefore allowed accurate spatial co-registration of the participant brains. We first selected a preliminary template, the brain of a healthy participant who was demographically representative of all participants in the study. We then similarity transformed (3 translations, 3 rotations, and a global scaling parameter), such that mutual information[12] was maximized, and then nonlinearly

warped using a fluid flow transformation[13] all participant brains to the initial template brain. The nonlinear warping established point-by-point correspondence across all points in the surface of the brain, which we used to compute the mean square distance of each participant's brain from the preliminary template. We then selected as the final template, the brain that was closest to the mean distance. This two-step procedure ensured that the final template brain was morphologically the most representative of all participant brains in our cohort. All participant brains were then first similarity co-registered and then nonlinearly warped to the final template.

Segmenting Brain Tissue The brain was isolated from nonbrain tissue in T1-weighted images by first applying the brain extract tool (BET)[14] and manually editing the connecting meninges. We then segmented brain tissue as gray matter, white matter, or cerebrospinal fluid by using a statistical classification scheme that yields Maximum-Likelihood (ML) estimates for tissue classes[15] based on an Expectation-Maximization (EM) algorithm.[16] This automated procedure simultaneously corrected for intensity non-uniformities and iteratively estimates tissue classes. Automatically segmented brain tissues then were then edited manually in all 3 views.

Surface Morphometry We mapped differences in brain morphology across cerebral surfaces for fine-grained subdivision of cerebral structures. Brain images were first rescaled to a common volume and then deformed using high-dimensional, nonlinear warping algorithms[13] such that grayscale intensities across the image match point-by-point those across a template brain image.[12] This matching provided a point-wise labeling of correspondences of the cortical surfaces across all brains in the sample. When the brains were unwarped, the distances of cortical surfaces from the template brain can be compared statistically across groups at each of these points along the surface. These distances were correlated with clinical, demographic, or behavioral variables of interest to assess their associations with local volumes at each point on the surface of the brain.

Measuring Cortical Thickness We computed cortical thickness measures in participant brains normalized to the final template brain, thereby scaling the cortical thickness measures for the whole brain volume (WBV). Cortical thickness measures for each participant brain were computed by first removing the cortical mantel from the similarity transformed brain, applying a 3D morphological operator that yielded the smallest distance from a point on the pial surface to the white matter surface. The WBV-scaled cortical thickness measures were then transferred from each participant brain to the corresponding points on the surface of the template brain.

DTI Processing We (1) visually inspected for motion or susceptibility artifacts; (2) corrected for phase-encoding distortions induced by eddy currents; (3) coregistered participant diffusion weighted (DW) images rigid body transformations;[12,17-21] and (4) corrected nonlinear geometric distortions in DWIs. We subsequently applied a model-free GQI method of Q-space imaging (QSI)[22] within DSI Studio for reconstructing the multi-shell data and to compute various DTI measures, including fractional anisotropy (FA), radial diffusivity (RD), average diffusivity coefficient (ADC), and axial diffusivity (AD). We also computed QSI-derived measures of edema and cellular infiltration[23], including (a) isotropic diffusion (**ISO**), an index for both restricted and unrestricted isotropic diffusion from edema; (b) non-restricted diffusion (**NRDI**), an index for non-restricted diffusion from edema, regardless of diffusion orientation; and (c) restricted diffusion (**RDI**), an index for cellular density and cell infiltration that is likely caused by inflammation. Diffusion tensor D was reconstructed by fitting an ellipsoid to diffusion weighted signal at each voxel. Positive definite property was ensured by decomposing tensor D as the product $D = A*A^T$, estimating matrix A, and then computed the tensor $\tilde{D} = \tilde{A} * \tilde{A}^T$. Positive definite tensor was factored into its eigenvalues $(\lambda_1, \lambda_2, \lambda_3)$ and eigenvectors (v_1, v_2, v_3), which we then used to calculate the scalar indices fractional anisotropy $FA = \frac{\sqrt{(\lambda_1-\lambda_2)^2+(\lambda_2-\lambda_3)^2+(\lambda_3-\lambda_1)^2}}{\sqrt{2*(\lambda_1^2+\lambda_2^2+\lambda_3^2)}}$, average diffusivity coefficient $ADC = \frac{1}{3}(\lambda_1 + \lambda_2 + \lambda_3)$, axial diffusivity $AD = \lambda_1$. And radial diffusivity $RD = \frac{1}{2}(\lambda_2 + \lambda_3)$. FA is a measure of directional diffusion of free water molecules, and is thought to represent integrity of tissue microarchitecture. ADC, on the other hand, is a measure average diffusion of free water molecules, and hence is though to represent tissue cell density.

The RDI index $\rho(L)$ was computed by averaging along all directions $\hat{u}$ on a unit sphere the spatial distribution $\Psi(\hat{u}, L)$ of the diffusing spins

$$\Psi\,(\hat{\boldsymbol{u}}, L) = L \sum_{q} W(\boldsymbol{q}) \cdot sinc(2\pi L \cdot |\boldsymbol{q}| \cdot < \hat{\boldsymbol{q}}, \hat{\boldsymbol{u}} >)$$

where $W(\boldsymbol{q})$ is the measured diffusion signal, L is the diffusion displacement, $\boldsymbol{q} = \gamma \boldsymbol{G} \delta / 2\pi$, γ is the gyromagnetic ratio, $\boldsymbol{G}$ is the direction and magnitude of the diffusion encoding gradient, δ is the duration of the diffusion encoding gradient, $\hat{\boldsymbol{q}}$ is the direction of the vector $\boldsymbol{q}$, and $|\boldsymbol{q}|$ is the magnitude of the vector $\boldsymbol{q}$. That is,

$$\rho(L) = \frac{1}{4\pi} \int_0^{\pi} \int_0^{2\pi} \Psi(\hat{u}, L) \cdot sin(\theta) \cdot d\phi \cdot d\theta$$

And the direction vector $\hat{u}$ is parameterized as $\hat{u} = (sin\theta \cdot cos\phi, sin\theta \cdot sin\phi, cos\theta)$.

We then coregistered voxelwise maps of various DTI and QSI-derived measures using rigid body transformation into the participant's anatomical MRI and then then nonlinear warping into the coordinate space of the template brain. The isotropic diffusion (ISO) is approximated as the minimum value of the spatial distribution $\Psi(\hat{u}, L)$ of the diffusing spins.[22] The NRDI index is computed as the difference $\rho(L_{max}) - \rho(L)$, where L_{max} is the maximum diffusion displacement L where spin density

$$\delta(L) = \sum_{\boldsymbol{q}} W(\boldsymbol{q}) \cdot sinc(2\pi L \cdot |\boldsymbol{q}|)$$

is non-negative.

Perfusion MRI Processing Perfusion images threshed to remove background noise and then were realigned to the first acquired volume using a rigid body transformation (3 rotations and 3 translations) such that mutual information[12] was maximized. The coregistered images were smoothed by applying a Gaussian kernel of 6mm FWHM (Full Width at Half Maximum) for improving signal-to-noise in the data. We then pairwise subtracted the smoothed control and smoothed labeled images and averaged the subtracted images across the imaging time series to compute the perfusion-weighted image. The quantitative regional cerebral blood flow (rCBF) map for each participant was computed from the perfusion-weighted image (ΔM) using a single compartment model as[24] $rCBF = \frac{6000 \cdot \Delta M \cdot e^{PLD/T_{1a}} \cdot e^{TE/T^*_{2a}}}{2\alpha \cdot \alpha_{inv} \cdot M_{0a} \cdot T_{1a}(1 - e^{-\tau/T_{1a}})}$, where post labeling delay (PLD) = 1,525 ms, equilibrium magnetization of arterial blood (M_{0a}), the longitudinal relaxation time of the arterial blood $(T_{1a}) = 1.8\ sec$, transverse relaxation time of the arterial blood $(T^*_{2a}) = 50\ ms$, labeling efficiency $(\alpha) = 0.8$, the correction for the labeling efficiency due to 2 background suppression pulses[25] $(\alpha_{inv}) = 0.83$. The equilibrium magnetization of the arterial blood M_{oa} is estimated by multiplying the equilibrium magnetization of the CSF M_{oCSF} with the blood-water partition coefficient of 0.76 ml water/ml water[26,27] and density of the brain tissue (1.05 g/ml).[28] The rCBF data were normalized into the coordinate space of the template space by using the localizer images that were acquired in the same slice locations as the rCBF data. We first used a rigid-body transformation to coregister localizer image and rCBF data to participant anatomical MR image. Subsequently, the similarity and nonlinear warping transformations[13] that normalized the anatomical image to the template brain were applied to normalize rCBF data into the template coordinate space. Regional blood flow is tightly coupled with local metabolic activity and, hence, is considered a marker for metabolism.

MR Spectroscopy Processing We analyzed the MRS data in the 3DiCSI software package (http://hatch.cpmc.columbia.edu/software.html) for visually assessing MRS spectra within the brain. We rejected spectra from further processing that had excessive noise, insufficient water suppression, distorted spectrum baseline, signal contamination from scalp lipids, linewidth broadening >12 Hz, unresolved choline

and creatine peaks at principal resonances, and incorrect placement of the outer volume suppression bands. We subsequently applied a least-squares estimation procedure for the automatic fitting of Gaussian-Lorentzian curves to frequency domain peaks for N-acetyl aspartate (NAA, 2.01 ppm), creatine (Cr, 3.01 ppm), and choline (Cho, 3.24 ppm). The area under the fitted peaks estimated the concentration of metabolites concentrations in the brain. NAA is considered as an index for the density of healthy neurons,[29] and is essential for signaling between neurons and oligodendrocytes.[30] Choline, an integral part of phospholipids in cellular lipid bilayer,[31] is not MRI visible when bound to membrane.[32] Choline peak therefore is used as a marker for structural integrity and tissue turnover.[33] We calculated background noise as the standard deviation of the part of the real spectrum free from metabolite signal, and generated a spectroscopic image for NAA as the ratio of peak area to background noise for each voxel, accounting for variations in receiver and transmitter gain. The average SNR for the NAA peak was > 200, suggesting an excellent acquisition of the MRS spectrum. This generated metabolite images on a 24x24 array in 6 slices through the brain at the MRS resolution of 10x10x12 mm^3.

The low spatial resolution metabolite images were resampled to the 1x1x1 mm^3 resolution of the T1-weighted images of the brain while correcting for the partial-volume (variable gray- *vs.* white-matter content across MPCSI voxels) and for dispersion of the MR signal into neighboring voxels using the MPCSI point spread function (PSF). The PSF was estimated by simulating MRS data on a 24x24 grid in k-space and then applying a Hamming window to spatially filter the simulated data. We segmented brain tissue as gray matter or white matter in the high-resolution T1-weighted MRI and similarity transformed this segmentation into the metabolite image. The gray- and white-matter components were then convolved with the PSF, thereby yielding fractions of gray and white matter (c_i^{GM}, c_i^{WM}) in each MPCSI voxel. The metabolite signal S_i in a voxel i was then modeled as metabolite levels in gray matter alone M_i^{GM} or white matter alone M_i^{WM} of that voxel as $S_i = c_i^{GM} * M_i^{GM} + c_i^{WM} * M_i^{WM} + \epsilon_i$, where ϵ_i is noise. Gray matter and white matter metabolite levels M_i^{GM}, M_i^{WM} in each voxel i were estimated by applying linear regression analysis to metabolite signal S_i and gray and white matter fraction c_i^{GM}, c_i^{WM} to the voxel i and its 4 immediate neighboring voxels in the MRS data. We trlinearly resampled metabolite levels from the low-resolution MPCSI to the high-resolution T1 during spatial normalization, which required coregistering and nonlinearly warping the MPCSI volume for each participant onto the T1 template brain. We then used the T1 image and high-resolution localizer to coregister each metabolite image to the template brain.

Statistical Analyses

All statistical analyses covaried for age and risk group. We did not covary for sex because only 2 patients and 2 healthy controls were female. Similarly, because our cohort included 5 SR and 11 HR patients, we did not assess the moderating effects of risk group on the progression of brain abnormalities, nor did we assess whether measures differed across risk groups. Findings did not change if risk group was included as a covariate, and therefore we present findings for analyses including this covariate.

Hypotheses were tested at each voxel either across the surface of the brain for anatomical MRI data or within the brain for ASL, DTI, and MRS data. We controlled for false positives in testing multiple hypotheses using topological False Discovery Rate (FDR)[34] at an FDR = 0.05. Findings that survived correction were color-coded and displayed on the International Consortium for Brain Mapping (ICBM) brain (http://www.loni.usc.edu/atlases/Atlas_Detail.php?atlas_id=5),[35] which has high tissue contrast and well-defined cortical sulci. We overlayed outlines for major sulci in green on the surface of the ICBM brain and provided Z coordinates for transverse slices in the Talairach atlas space[36] to aid localization of findings.

A Priori Hypotheses Testing

(1) We tested the a priori hypothesis that brain abnormalities will progressively worsen by applying a univariate repeated measures analysis. We evaluated statistical significance of the Dx-by-time effect on changes in brain measures over time – i.e., whether brain measures changed differentially in patients from controls:

$$Brain_i = \beta_0 + \beta_1 * Age^b + \beta_2 * Dx + \beta_3 * Time_i + \beta_4 * Dx * Time + \epsilon_1$$

where $Brain_i$ is an MRI-derived repeated measure at either pre-methotrexate baseline or year 1, Age^b is age at baseline, $Dx = 1$ for patients and 0 for healthy controls, and $Time = 0$ for baseline and 1 for year 1.

To understand significant interactions, we subsequently evaluated how brain differences progressed separately in patients and controls from baseline to year 1 using a univariate repeated measures analysis.

$$Brain_i = \beta_0 + \beta_1 * Age^b + \beta_2 * Time_i + \epsilon_2$$

(2) We tested our a priori hypothesis that the worsening of brain abnormalities will be associated with higher doses of intrathecal methotrexate separately from (a) baseline to week 9, (b) week 9 to week 22, and (c) week 22 to year 1. We first assessed whether MRI-derived brain measures in patients changed between these timepoints and, if so, whether those changes were associated with then number of IT-MTX doses. Significant associations of the number of IT-MTX dose with changes in brain measures would suggest that brain changes likely were a consequence of IT-MTX administration. We did not control for intravenous MTX for the week 9 to week 22 analyses because all HR patients received the same amounts of HD-MTX, and all SR patients received the same doses of Capizzi MTX. Doses of intravenous MTX therefore were confounded with ALL risk. We did, however, control for age and risk in our models.

We used a univariate repeated measures analysis to test whether patients had brain injuries that progressed between these time points

$$Brain_i = \beta_0 + \beta_1 * Age^b + \beta_2 * Risk + \beta_3 * Time_i + \epsilon_3$$

where $Brain_i$ is brain measure at the two time points (a – c above); Age^b is the age at baseline; and $Risk = 1$ for HR patients and 0 for SR patients.

Within the patient group alone, we used a univariate, repeated measures analyses to test whether the progression of brain abnormalities was associated with IT-MTX doses separately from (a) baseline to week 9, (b) week 9 to week 22, and (c) week 22 to year 1.

$$Brain_i = \beta_0 + \beta_1 * Age^b + \beta_2 * Risk + \beta_3 * aMTX + \beta_4 * (MTX_i - aMTX) + \epsilon_4$$

where $Brain_i$ is brain measure at the two time points (a – c above); Age^b is the age at baseline; $Risk = 1$ for HR patients and 0 for SR patients; $aMTX$ is the average of doses at (a) baseline and week 9, (b) week 9 and week 22, or (c) week 22 and year 1; and $(MTX_i - aMTX)$ is change in MTX dose for each patient.

Secondary Analyses

(3) Progression of brain abnormalities can be modulated by whether patients relative to healthy controls have abnormalities as a consequence of acute illness. We therefore tested the hypothesis that patients relative to controls have brain abnormalities at pre-methotrexate baseline by applying a multiple linear regression analysis:

$$Brain^b = \beta_0 + \beta_1 * Age^b + \beta_2 * Dx + \epsilon_5$$

where $Brain^b$ is an MRI-derived brain measure, Age^b is participant age at the time of baseline scan, and $Dx = 1$ for patients and 0 for healthy controls. The p-value for regression coefficient β_2 assessed statistically significant of differences in patients relative to controls.

(4) Although baseline assessments were collected before intrathecal or systemic methotrexate, patients had received a single dose of intrathecal Cytarabine and daily doses of either dexamethasone or prednisone, which may induce baseline abnormalities in patients. We therefore converted[37,38] prednisone doses to equivalent dexamethasone doses and assessed within patients alone whether cumulative doses of corticosteroids received were associated with MRI-derived brain measures at the time of baseline scan.

$$Brain^b = \beta_0 + \beta_1 * Age^b + \beta_2 * Steroid + \epsilon_6$$

where $Brain^b$ is an MRI-derived brain measure, Age^b is participant age at the time of baseline scan, and $Steroid$ is the cumulated dose of corticosteroid received at the time of baseline scan.

Tertiary Analyses

(5) To understand whether baseline cognitive deficits in patients were due to brain abnormalities or due to acute illness disrupting those associations, we first used multiple linear regression analysis to assess how brain measures were associated with performance speed index (PSI) separately in healthy controls and in patients,

$$Brain^b = \beta_0 + \beta_1 * Age^b + \beta_2 * PSI + \epsilon_7$$

and evaluated the statistical significance of the coefficient β_2. We then tested whether this association in patients differed from that in healthy controls by computing the multiple linear regression model:

$$Brain^b = \beta_0 + \beta_1 * Age^b + \beta_2 * PSI + \beta_3 * Dx + \beta_4 * PSI * Dx + \epsilon_8$$

and computed the statistical significance of the coefficient β_4.

(6) Patients with HR ALL during the interim maintenance phase were treated with leucovorin rescue. We assessed whether leucovorin rescue had neuroprotective effects in HR patients:

$$Brain_i = \beta_0 + \beta_1 * Age^b + \beta_2 * MTX_i + \beta_3 * Leuc_i + \epsilon_9$$

where $Brain_i$ is brain measure at week 9 or week 22, MTX_i is the number of doses at week 9 or week 22, and $Leuc_i = 0$ for week 9 and cumulative leucovorin dose for week 22. All HR patients received the same dose of HD-MTX, and hence we did not include it as a covariate.

(7) We used structural equation modeling (SEM) within Mplus Statistical Software to assess whether changes among various MRI measures were causally interrelated.[39] For these analyses, we first generated separate masks of brain regions where cortical thickness, N-acetyl aspartate (NAA) level, rCBF level, or FA values changed significantly with time within patients. These masks were then applied to compute average values of CT, NAA, rCBF, and FA for each participant. We then regressed out age and risk effects to generate residual CT, NAA, rCBF, and FA values at each of the 4 timepoints: baseline, week 9, week 22, and year 1. We then modeled the causal influences in longitudinal panel data while modeling the autoregressive relationships among MR

modality measures and the actual within-person relationships over time using a random intercept, cross-lagged panel model (RI-CLPM).[40] The RI-CLPMs did not converge, however, likely because data were available at all time points for only 13 patients. We therefore applied SEMs to the regressed data within each time point to assess the causal relationships between brain measures and to assess whether those causal relations were disrupted by chemotherapy over time. We specified an SEM using the following regression equations:

$$CT_i = \alpha_{CT_i} + \rho_{CT_i FA_i} \cdot FA_i + \rho_{CT_i NAA_i} \cdot NAA_i + \epsilon_{CT_i}$$
$$rCBF_i = \alpha_{rCBF_i} + \rho_{rCBF_i CT_i} \cdot CT_i + \epsilon_{rCBF_i}$$

where $i = 1, 2, 3, 4$ for baseline, week 9, week 22, and year 1, respectively; α and ρ are within-timepoint model parameters to be estimated; CT_i are cortical thickness measures at time i; FA_i are FA values at time i; NAA_i are NAA values at time i; $rCBF_i$ are rCBF at time i; and ϵ are the error terms. The optimal parameters are estimated by maximizing the Wishart Likelihood function (MLW) using the least-squares programming (SLSQP) optimization method. The p-values for the estimated parameters are computed assuming that the parameters are Gaussian distributed under the null hypothesis. We also computed the goodness of the fitted model by computing the Chi-Square statistic that tests the null hypothesis that the variances, covariances, and means suggested by the model do not differ from the population values.[41] The null hypothesis is rejected when the chi-square test statistic is statistically significant – i.e., when the specified model does not accurately model the population values. The chi-square test, however, is over-powered and, hence, typically rejects the null hypothesis. We therefore also computed the absolute fit index of root mean square error of approximation (RMSEA), and used RMSEA values < 0.05 as a good fit of the specified model to population data.[42,43]

eRESULTS

Participant Recruitment and Retention

We approached 78 consecutive patients with newly diagnosed ALL at CHLA, of whom 71 were eligible but only 19 patients consented to participate in the study (**Fig.1**): 5 patients with SR ALL, who completed study assessments at all 4 time points; 14 patients with HR ALL. Most patients or their parents declined participation because of the acute, life-threatening nature of ALL, the overall level of fatigue and weakness of the youth, and their reluctance to add research assessments to an already high burden of clinical assessments upon the initial ALL diagnosis. Patients who did consent, however, seemed highly motivated and continued participation for the entire 1-year follow-up. Only three HR patients dropped out after baseline assessment because they were anxious during the MRI. One HR patient in the consolidation phase developed a brief stroke-like syndrome, which was classified as grade 1 neurotoxicity. That patient recovered and continued participation. Three more HR patients were refractory to chemotherapy or developed early relapse and therefore were transferred to other salvage treatments after the week 22 assessments. We therefore had data in 11 HR patients at 3 time points, and in 8 HR patients and 5 SR patients at all 4 time points (**Fig.1**). Each analysis included only participants who had complete data for that analysis. We also collected MRI data and neuropsychological assessments in 20 age- and sex-matched healthy controls at baseline and one year later in 15 of those controls.

Baseline Assessments

Baseline assessments were collected within the first week of chemotherapy initiation prior to patients receiving intrathecal or systemic methotrexate. Acquiring assessments earlier was not feasible as chemotherapy was initiated within 3 days of the initial diagnosis in most patients (**eFig.2**). Patients prior to pre-MTX baseline assessments therefore had received a single 70 mg/m^2 dose of intrathecal cytarabine and daily doses of either 60 mg/m^2 prednisone for HR ALL or 6 mg/m^2 dexamethasone for SR ALL.

Corticosteroid Associations with Brain Measures

Cumulative steroid dose was not associated with WM measures of local volumes, ADC and FA indices for microstructure integrity, or NAA (**eFig.3**) in brain regions where patients had baseline abnormalities (**Fig.2**). Steroid dose however was positively associated with local volumes of WM in prefrontal and occipital lobes of the right hemisphere and in superior parietal regions of the left hemisphere (**eFig.3**). Furthermore, higher cumulative doses were associated with thicker cortex, and increased rCBF but lower FA values in GM of the brain (**eFig.3**).

Performance Speed Associations with Brain Measures

At baseline healthy participants with higher Performance Speed Index (PSI) had thicker cortex in the prefrontal and occipital lobes, higher WM volumes in superior prefrontal, parietal, and occipital cortices, higher density of healthy neurons, lower rCBF, and lower ADC but higher FA in WM (**eFig.4A**). These associations were generally conserved in patients at pre-treatment baseline: patients with higher PSI had thicker cortex across lateral and mesial frontal and parietal lobes, higher WM volumes especially in the frontal and temporal lobes, lower rCBF, lower NAA in the posterior WM but higher NAA in anterior and dorsal WM, higher ADC in superior but lower in inferior GM, and higher FA in WM but lower FA in GM of the cortex (**eFig.4B**). Associations of cortical thickness, local volumes of WM, and rCBF with PSI in healthy did not differ from those in patients (**eFig.4C**). Associations of FA and ADC with PSI in patients did differ from those in healthy in some cortical GM, and superior WM of the brain (**eFig.4C**).

Leucovorin Rescue

Higher doses of leucovorin rescue were associated with cortical thickening in the prefrontal and occipital lobes, local WM volumes increases in the somatosensory cortex, rCBF decreases in cortical GM and increases in WM, NAA increases in cortical mantel, ADC increases in ventral GM but decreases in prefrontal cortex, and FA decreases in cortical GM but increases in WM (**eFig.5**).

Baseline to Year 1 Changes in Brain Measures in Healthy Controls

Healthy controls showed decreases in cortical thickness that reached statistical significance in small regions across the entire brain (**eFig.6**). rCBF values decreased in cortical gray matter and in the caudate nucleus (**eFig.6**). Local WM volumes, NAA, and ADC measures did not change from baseline to year 1. FA values in healthy controls, however, decreased in post thalamic radiations (**eFig.6**).

Cross-Sectional Relationships among Multimodal Brain Measures

Brain measures across various MRI modalities were positively correlated at all time points: increases in FA or NAA values were associated with increases in cortical thickness, which in turn was associated with increases in rCBF. However, the strength of these predictive associations varied across time and differed between hemispheres. The SEM model fitted to the baseline multimodal MRI data accurately modeled population variances, covariances, and means ($RMSEA = 0.0$; $\chi^2 = 0.74, p = 0.7$); changes in FA ($p = 0.003$) and NAA ($p = 0.05$) predicted changes in cortical thickness, which subsequently predicted changes in rCBF ($p = 0.04$) (**eFig.7**, *Base*). At week 9, even though FA values significantly predicted cortical thickness ($p < 0.0001$), which in turn predicted rCBF values ($p = 0.018$), the fitted model poorly modeled week 9 data ($RMSEA =$

0.25; $\chi^2 = 3.9, p = 0.14$) because NAA values did not significantly ($p = 0.65$) predict cortical thickness (**eFig.7**, *w9*). At week 22, the fitted model failed to accurately predict the population data ($RMSEA = 0.19$; $\chi^2 = 3.1, p = 0.21$), even though FA significantly predicted cortical thickness ($p = 0.001$), which in turn significantly predicted rCBF values ($p = 0.005$) (**eFig.7**, *w22*). By year 1, the fitted SEM model accurately predicted causal relation among brain measures ($RMSEA = 0.0$; $\chi^2 = 0.005, p = 0.99$); FA ($p = 0.02$) and NAA ($p = 0.004$) accurately predicted cortical thickness, which in turn accurately predicted rCBF values ($p = 0.007$) (**eFig.7**, *Y1*).

Changes in AD and RD

AD and RD values did not change from baseline to week 9, nor from week 9 to week 22 (**eFigs. 11&12,** *left panel*). Because RD and AD values did not change, we were unable to detect their associations with IT-MTX doses received (**eFigs. 11&12,** *right panel*). However from week 22 to year 1, both RD and AD values declined significantly within WM of the brain, especially in the anterior and posterior corona radiata (aCR and pCR), external capsule (EC), **SS**, superior longitudinal fasciculus (SLF), and superior corona radiata (sCR) (**eFigure13,** *left panel*).. These changes in RA and AD were significantly associated with increasing IT-MTX doses, suggesting that WM changes indexed by RD and AD are likely consequences of the IT-MTX (**eFigure13,** *right panel*).

Changes AD and RD values are consistent with those for ADC and FA values (**Figs. 3,4,&5**): the ADC values in the same WM pathways declined from week 22 to year 1 because ADC is the average of the diffusivity values along the three eigendirections of a diffusion tensor. FA values, however, increased over that time frame, suggesting that RD values declined more than AD values, causing a relative increase in the directional diffusion of water. In contrast, IT-MTX associated significantly more across spatially extensive WM regions with changes in AD and RD values (**eFigs. 11,12,& 13**) than with ADC or FA values (**Figs. 3,4,&5**), suggesting that changes in AD and RD could possibly provide more sensitive measures for assessing effects of IT-MTX on WM microstructure.

Changes in ISO, NRDI, and RDI Measures

Analyses of ISO, NRDI, and RDI showed that these measures (a) did not change from baseline to week 9, (b) declined from week 9 to week 22, especially in GM regions, and (c) did not change from week 22 to year 1 (**eFigs. 11,12,& 13**). Decreases in ISO, NRDI, and RDI from week 9 to week 22 suggests local edema and cellular infiltration declined significantly from week 9 to week 22 in GM regions. The decreasing edema in GM may have contributed to cortical thinning. On the other hand, decreasing ADC, RD, and AD, and the increase in FA within WM from week 22 to year 1, possibly is derived from increasing axonal myelination and repair during that time.

eDISCUSSION

Relationship to Prior Studies

Our findings of brain abnormalities at baseline are consistent with one prior study that reported lower functional connectivity within 1 week of beginning chemotherapy.[44] Our findings that brain abnormalities are present in patients without overt clinical neurotoxicity are consistent with those from a prior study that documented WM hyperintensities in patients without overt neurotoxicity.[45] Our findings that WM abnormalities normalize by year 1 are consistent with prior studies reporting transient WM hyperintensities during treatment[46-48] and

declining water diffusivities over time.[49] Our findings that patients had cognitive impairments at year 1 are consistent with a prior study that reported enduring attentional problems in ALL patients,[50] and cognitive deficits during and post treatment.[48,51] Furthermore, our findings that cognitive deficits persisted while WM normalized by year 1 is consistent with prior findings that WM hyperintensities did not associate significantly with cognitive performance.[46,48]

Some of our findings, however, differed in important ways from those of prior studies. We found that patients within one week of treatment had significant gray and white matter abnormalities and neurocognitive deficits compared to controls, whereas past studies have variously reported that patients at various points in treatment either did not have white matter hyperintensities,[47,48,51] had normal WM integrity as indexed by DTI measures,[51] had normal brain metabolite levels (NAA/Cho and NAA/Cr ratios),[51] or did not have cognitive deficits.[44,51] One prior study reported that brain metabolite concentrations (NAA/Cho ratio) declined transiently but normalized over time, in contrast to our finding that low metabolite levels at baseline declined further over the year.[47] Finally, in our study patients had cognitive impairments that persisted but did not worsen from baseline to year 1, whereas one prior study reported that working memory and performance speed declined from baseline to the end of chemotherapy.[51]

Our differing baseline findings from those in prior studies may be attributable to differing patient demographics: patients in our cohort were predominantly male Hispanics, whereas patients in other studies were mostly white Caucasians or Asians. The absence of abnormalities at baseline in prior studies could also be attributable to different procedures used to process and statistically analyze MRI data. Some prior studies, for example, visually assessed WM for hyperintensities,[47,48,51] which likely would miss subtle disturbances in tissue intensities that our procedures detected using more precise quantification. Moreover, some prior studies assessed DTI measures at a small number of prespecified brain regions,[51] and therefore may have missed regions with abnormal measures. Finally, the absence of metabolite abnormalities in prior studies may be attributable to use of metabolite ratios, such as NAA/Cho or NAA/Cr.[51] The ratio of variables amplifies noise and reduces statistical power to detect differences. Use of a ratio would also fail to detect metabolite abnormalities if NAA, Cho, and Cr levels change proportionally, which previously has been shown to be true.[52] Similarly, normalization of NAA/Cho levels by year 1 may be attributable to use of a ratio rather than true normalization of brain metabolites.[47] Finally, the worsening of cognitive deficits from baseline to completion of chemotherapy in one prior study[51] could be attributable to the development of overt neurotoxicity in some of those participants, whereas none of our patients exhibited neurotoxicity.

Brain Networks Subserving Attention and Working Memory

Our MRI data showed that gray matter abnormalities were located in the frontal lobe, anterior cingulate cortex, posterior cingulate cortex, cuneus, temporal lobe, insular cortex, and the striatum -- brain regions that support attention and higher-order cognition.[53-65] WM abnormalities, in contrast, were located in the superior longitudinal fasciculus, corona radiata, external capsule, cingulum bundle, and corpus callosum – WM tracts that interconnect those gray matter regions. Faster processing speeds in healthy controls were associated with thicker cortices, larger WM volumes, higher NAA, lower ADC, and higher FA values (**eFig.2**). In these same brain regions, patients relative to controls had thinner cortices, higher ADC, lower FA, lower NAA, and higher WM local volumes (**Fig.2**), suggesting that their attentional and working memory deficits may be a consequence of brain abnormalities. The cognitive abnormalities at pre-MTX baseline could be attributable to emotional stress and fatigue, but the persistence of these abnormalities at 1 year suggest instead that they are true deficits at baseline, likely consequences of illness.

MTX Toxicity to Mature, Post-Mitotic Cells

Both MTX and cytarabine hinder DNA replication and hence are cytotoxic to immature oligodendrocyte precursor cells (OPCs) and neural progenitor cells (NPC). Chemotherapy drugs can also induce cell death in post-mitotic oligodendrocytes[66] and neurons, however, by damaging their DNA,[67-69] which can be cytotoxic.[67] Chemotherapy also reduces dendritic branching and spine density.[70] MTX has also been shown to induce apoptosis in astrocytes by disrupting folate metabolism.[71,72] Chemotherapy medications also reduce transcription and translation[73] of both brain derived neurotrophic factors (BDNF),[74,75] which is essential for neuronal health, growth, and differentiation,[76,77] and genes involved in protecting neurons from oxidant stress and apoptosis.[78] Thus, chemotherapy medications cause cell death and suppress cell division[66] and may thereby produce the neurological and cognitive deficits[79] reported in survivors of childhood ALL.

Structural Equation Modeling

The fitted SEM accurately modeled the interrelationships of MRI data at baseline. The fit was poor at week 9 and week 22, however, then was restored by year 1. Model fits were poor because at week 9 and week 22 because NAA values were not predictive of cortical thickness (**eFig.5**). These SEM analyses suggest that the MRI findings are fundamentally determined by reductions in neuronal density, as indexed by lower NAA levels, and by cerebral edema (by extension, also disruption of the blood brain barrier) and myelin loss, as represented by altered FA and ADC. By year 1, local WM volumes, FA, and ADC values normalized, likely as cerebral edema subsided, thereby restoring the predictive associations between neuronal density and cortical thickness.

eREFERENCES

1 Hunger, S. P. et al. Children's Oncology Group's 2013 Blueprint for Research: Acute Lymphoblastic Leukemia. *Pediatr Blood Cancer* **60**, 957-963 (2013).

2 Mitchell, H. R. et al. Prospective, Longitudinal Assessment of Quality of Life in Children from Diagnosis to 3 Months Off Treatment for Standard Risk Acute Lymphoblastic Leukemia: Results of Children's Oncology Group Study Aall0331. *Int J Cancer* **138**, 332-339 (2016).

3 Howard, S. C., McCormick, J., Pui, C. H., Buddington, R. K. & Harvey, R. D. Preventing and Managing Toxicities of High-Dose Methotrexate. *Oncologist* **21**, 1471-1482 (2016).

4 Wechsler, D. *Wechsler Preschool and Primary Scale of Intelligence-Fourth Edition (Wppsi-Iv)* (Pearson, Psychological Corporation, 2012).

5 Raggio, D. J., Scattone, D. & May, W. Relationship of the Kaufman Brief Intelligence Test Second Edition and the Wechsler Abbreviated Scale of Intelligence in Children Referred for Adhd. *Psychol Rep* **106**, 513-518 (2010).

6 Wechsler, D. *Wechsler Intelligence Scale for Children-Fifth Edition. Administration and Scoring Manual* (Pearson, Bloomington, MN, 2014).

7 Wechsler, D. *Wechsler Adult Intelligence Scale-Fourth Edition (Wais-Iv)* (Pearson, Bloomington, MN, 2008).

8 Kaufman, J. et al. Schedule for Affective Disorders and Schizophrenia for School-Age Children-Present and Lifetime Version (K-Sads-Pl): Initial Reliability and Validity Data. *J Am Acad Child Adolesc Psychiatry* **36**, 980-988 (1997).

9 Poznanski, E. O., Freeman, L. N. & Mokros, H. B. Childrens Depression Rating-Scale - Revised (September 1984). *Psychopharmacology Bulletin* **21**, 979-989 (1985).

10 DuPaul, G. J. Parent and Teacher Ratings of Adhd Symptoms: Psychometric Properties in a Community-Based Sample. *J Clin Child Psychology* **20**, 245-253 (1991).

11 Constantino, J. *The Social Responsiveness Scale.*, Western Psychological Service., (2002).

12 Viola, P. & Wells, W. M. in *Fifth International Conference on Computer Vision.* 16-23.

13 Christensen, G. E., Joshi, S. C. & Miller, M. I. Volumetric Transformation of Brain Anatomy. *IEEE Transactions on Medical Imaging* **16**, 1369-1383 (1997).

14 Smith, S. M. Fast Robust Automated Brain Extraction. *Human Brain Mapping* **17**, 143-155 (2002).

15 Wells, W. M., Grimson, W. E. L., Kikinis, R. & Jolesz, F. A. Adaptive Segmentation of Mri Data. *IEEE Trans Med Img* **15**, 429-442 (1996).

16 Dempster, A. P., Laird, N. M. & Rubin, D. B. Maximum Likelihood from Incomplete Data Via the Em Algorithm. *J Royal Stat Soc* **39**, 1-38 (1977).

17 Woods, R. P., Grafton, S. T., Holmes, C. J., Cherry, S. R. & Mazziotta, J. C. Automated Image Registration: I. General Methods and Intrasubject, Intramodality Validation. *Journal of Computer Assisted Tomography* **22**, 139-152 (1998).

18 Woods, R. P., Grafton, S. T., Watson, J. D., Sicotte, N. L. & Mazziotta, J. C. Automated Image Registration: Ii. Intersubject Validation of Linear and Nonlinear Models. *Journal of Computer Assisted Tomography* **22**, 153-165 (1998).

19 Friston, K. J. et al. Spatial Registration and Normalization of Images. *Human Brain Mapping* **3**, 165-189 (1995).

20 Collignon, A. et al. Automated Multimodality Image Registration Using Information Theory. *Proceedings of the 14th International Conference*, 263-274 (1995).

21 Studholme, C., Hill, D. L. G. & Hawkes, D. J. An Overlap Invariant Entropy Measure of 3d Medical Image Alignment. *Pattern Recognition* **32**, 7186 (1999).

22 Yeh, F. C., Wedeen, V. J. & Tseng, W. Y. Generalized Q-Sampling Imaging. *IEEE Trans Med Imaging* **29**, 1626-1635 (2010).

23 Yeh, F. C., Liu, L., Hitchens, T. K. & Wu, Y. L. Mapping Immune Cell Infiltration Using Restricted Diffusion Mri. *Magn Reson Med* **77**, 603-612 (2017).

24 Alsop, D. C. et al. Recommended Implementation of Arterial Spin-Labeled Perfusion Mri for Clinical Applications: A Consensus of the Ismrm Perfusion Study Group and the European Consortium for Asl in Dementia. *Magnetic Resonance in Medicine* **73**, 102-116 (2015).

25 Mutsaerts, H. J. M. M. et al. Inter-Vendor Reproducibility of Pseudo-Continuous Arterial Spin Labeling at 3 Tesla. *PLoS One* **9** (2014).

26 Herscovitch, P. & Raichle, M. E. What Is the Correct Value for the Brain Blood Partition-Coefficient for Water. *Journal of Cerebral Blood Flow and Metabolism* **5**, 65-69 (1985).

27 Heijtel, D. F. R. et al. Accuracy and Precision of Pseudo-Continuous Arterial Spin Labeling Perfusion During Baseline and Hypercapnia: A Head-to-Head Comparison with O-15 H2o Positron Emission Tomography. *NeuroImage* **92**, 182-192 (2014).

28 Chalela, J. A. et al. Magnetic Resonance Perfusion Imaging in Acute Ischemic Stroke Using Continuous Arterial Spin Labeling. *Stroke* **31**, 680-687 (2000).

29 Zhu, H. & Barker, P. B. Mr Spectroscopy and Spectroscopic Imaging of the Brain. *Methods Mol Biol* **711**, 203-226 (2011).

30 Moffett, J. R., Ross, B., Arun, P., Madhavarao, C. N. & Namboodiri, A. M. N-Acetylaspartate in the Cns: From Neurodiagnostics to Neurobiology. *Prog Neurobiol* **81**, 89-131 (2007).

31 Michel, V., Yuan, Z., Ramsubir, S. & Bakovic, M. Choline Transport for Phospholipid Synthesis. *Exp Biol Med (Maywood)* **231**, 490-504 (2006).

32 Nelson, S. J. Multivoxel Magnetic Resonance Spectroscopy of Brain Tumors. *Mol Cancer Ther* **2**, 497-507 (2003).

33 Rae, C. D. A Guide to the Metabolic Pathways and Function of Metabolites Observed in Human Brain 1h Magnetic Resonance Spectra. *Neurochem Res* **39**, 1-36 (2014).

34 Chumbley, J., Worsley, K., Flandin, G. & Friston, K. Topological Fdr for Neuroimaging. *Neuroimage* **49**, 3057-3064 (2010).

35 Mazziotta, J. et al. A Probabilistic Atlas and Reference System for the Human Brain: International Consortium for Brain Mapping (Icbm). *Philosophical Transactions of the Royal Society of London - Series B: Biological Sciences* **356**, 1293-1322 (2001).

36 Talairach, J. & Tournoux, P. *Co-Planar Stereotaxic Atlas of the Human Brain: 3-Dimensional Proportional System: An Approach to Cerebral Imaging; 1st Edition* (Thieme, 1988).

37 Webb, R. & Singer, M. *Oxford Handbook of Critical Care* (Oxford University Press, 2005).

38 Meikle, A. W. & Tyler, F. H. Potency and Duration of Action of Glucocorticoids. Effects of Hydrocortisone, Prednisone and Dexamethasone on Human Pituitary-Adrenal Function. *Am J Med* **63**, 200-207 (1977).

39 Muthén, L. K. & Muthén, B. O. *Mplus User's Guide. Eighth Edition.* (Muthén & Muthén, 1997-2017).

40 Hamaker, E. L., Kuiper, R. M. & Grasman, R. P. P. P. A Critique of the Cross-Lagged Panel Model. Psychological Methods. *20* **1**, 102-116 (2015).

41 West, S. G., Taylor, A. B. & Wu, W. in *Handbook of Structural Equation Modeling* (Hoyle, R. ed.) 209-231 (2012).

42 Hu, L. T. & Bentler, P. M. Cutoff Criteria for Fit Indexes in Covariance Structure Analysis: Conventional Criteria Versus New Alternatives. *Structural Equation Modeling: A Multidisciplinary Journal* **6**, 1-55 (1999).

43 Browne, M. W. & Cudeck, R. Alternative Ways of Assessing Model Fit. *Sociological Methods & Research* **21**, 230-258 (1992).

44 Hu, Z. et al. Altered Brain Function in New Onset Childhood Acute Lymphoblastic Leukemia before Chemotherapy: A Resting-State Fmri Study. *Brain Dev* **39**, 743-750 (2017).

45 Bhojwani, D. et al. Methotrexate-Induced Neurotoxicity and Leukoencephalopathy in Childhood Acute Lymphoblastic Leukemia. *J Clin Oncol* **32**, 949-959 (2014).

46 Paakko, E. et al. White Matter Changes on Mri During Treatment in Children with Acute Lymphoblastic Leukemia: Correlation with Neuropsychological Findings. *Med Pediatr Oncol* **35**, 456-461 (2000).

47 Chu, W. C. et al. White Matter and Cerebral Metabolite Changes in Children Undergoing Treatment for Acute Lymphoblastic Leukemia: Longitudinal Study with Mr Imaging and 1h Mr Spectroscopy. *Radiology* **229**, 659-669 (2003).

48 Wilson, D. A. et al. Transient White Matter Changes on Mr Images in Children Undergoing Chemotherapy for Acute Lymphocytic Leukemia: Correlation with Neuropsychologic Deficiencies. *Radiology* **180**, 205-209 (1991).

49 Ramli, N. et al. Assessing Changes in Microstructural Integrity of White Matter Tracts in Children with Leukaemia Following Exposure to Chemotherapy. *Pediatr Radiol* **50**, 1277-1283 (2020).

50 Jacola, L. M. et al. Longitudinal Assessment of Neurocognitive Outcomes in Survivors of Childhood Acute Lymphoblastic Leukemia Treated on a Contemporary Chemotherapy Protocol. *J Clin Oncol* **34**, 1239-1247 (2016).

51 Wei, K. et al. An Observational Mri Study of Methotrexate-Treated Children with Acute Lymphoblastic Leukemia in Remission and Subtle Cognitive Decline. *Quant Imaging Med Surg* **12**, 2474-2486 (2022).

52 Baron Nelson, M. et al. Maturation of Brain Microstructure and Metabolism Associates with Increased Capacity for Self-Regulation During the Transition from Childhood to Adolescence. *J Neurosci* **39**, 8362-8375 (2019).

53 Niendam, T. A. et al. Meta-Analytic Evidence for a Superordinate Cognitive Control Network Subserving Diverse Executive Functions. *Cogn Affect Behav Neurosci* **12**, 241-268 (2012).

54 Rowe, J. B., Toni, I., Josephs, O., Frackowiak, R. S. & Passingham, R. E. The Prefrontal Cortex: Response Selection or Maintenance within Working Memory? *Science* **288**, 1656-1660 (2000).

55 du Boisgueheneuc, F. et al. Functions of the Left Superior Frontal Gyrus in Humans: A Lesion Study. *Brain* **129**, 3315-3328 (2006).

56 Japee, S., Holiday, K., Satyshur, M. D., Mukai, I. & Ungerleider, L. G. A Role of Right Middle Frontal Gyrus in Reorienting of Attention: A Case Study. *Front Syst Neurosci* **9**, 23 (2015).

57 Kapur, S. et al. Neuroanatomical Correlates of Encoding in Episodic Memory: Levels of Processing Effect. *Proc Natl Acad Sci U S A* **91**, 2008-2011 (1994).

58 Kubler, A., Dixon, V. & Garavan, H. Automaticity and Reestablishment of Executive Control-an Fmri Study. *J Cogn Neurosci* **18**, 1331-1342 (2006).

59 Goel, V., Gold, B., Kapur, S. & Houle, S. The Seats of Reason? An Imaging Study of Deductive and Inductive Reasoning. *Neuroreport* **8**, 1305-1310 (1997).

60 Druks, J. *Contemporary and Emergent Theories of Agrammatism: A Neurolinguistic Approach* (Routledge, 2016).

61 Woodward, T. S., Ruff, C. C. & Ngan, E. T. Short- and Long-Term Changes in Anterior Cingulate Activation During Resolution of Task-Set Competition. *Brain Res* **1068**, 161-169 (2006).

62 Carter, C. S., Mintun, M. & Cohen, J. D. Interference and Facilitation Effects During Selective Attention: An H215o Pet Study of Stroop Task Performance. *Neuroimage* **2**, 264-272 (1995).

63 Buckner, R. L., Andrews-Hanna, J. R. & Schacter, D. L. The Brain's Default Network: Anatomy, Function, and Relevance to Disease. *Ann N Y Acad Sci* **1124**, 1-38 (2008).

64 Van Etten, R. A. Aberrant Cytokine Signaling in Leukemia. *Oncogene* **26**, 6738-6749 (2007).

65 Drevets, W. C., Savitz, J. & Trimble, M. The Subgenual Anterior Cingulate Cortex in Mood Disorders. *CNS Spectr* **13**, 663-681 (2008).

66 Dietrich, J., Han, R., Yang, Y., Mayer-Proschel, M. & Noble, M. Cns Progenitor Cells and Oligodendrocytes Are Targets of Chemotherapeutic Agents in Vitro and in Vivo. *J Biol* **5**, 22 (2006).

67 Maynard, S., Fang, E. F., Scheibye-Knudsen, M., Croteau, D. L. & Bohr, V. A. DNA Damage, DNA Repair, Aging, and Neurodegeneration. *Cold Spring Harb Perspect Med* **5** (2015).

68 Patel, J. et al. DNA Damage and Mitochondria in Cancer and Aging. *Carcinogenesis* **41**, 1625-1634 (2020).

69 Hoeijmakers, J. H. DNA Damage, Aging, and Cancer. *N Engl J Med* **361**, 1475-1485 (2009).

70 Zhou, W., Kavelaars, A. & Heijnen, C. J. Metformin Prevents Cisplatin-Induced Cognitive Impairment and Brain Damage in Mice. *PLoS One* **11**, e0151890 (2016).

71 Gregorios, J. B. & Soucy, D. Effects of Methotrexate on Astrocytes in Primary Culture: Light and Electron Microscopic Studies. *Brain Res* **516**, 20-30 (1990).

72 Shao, Y., Tan, B., Shi, J. & Zhou, Q. Methotrexate Induces Astrocyte Apoptosis by Disrupting Folate Metabolism in the Mouse Juvenile Central Nervous System. *Toxicol Lett* **301**, 146-156 (2019).

73 Geraghty, A. C. et al. Loss of Adaptive Myelination Contributes to Methotrexate Chemotherapy-Related Cognitive Impairment. *Neuron* **103**, 250-265 e258 (2019).

74 Mustafa, S., Walker, A., Bennett, G. & Wigmore, P. M. 5-Fluorouracil Chemotherapy Affects Spatial Working Memory and Newborn Neurons in the Adult Rat Hippocampus. *Eur J Neurosci* **28**, 323-330 (2008).

75 Park, H. S. et al. Physical Exercise Prevents Cognitive Impairment by Enhancing Hippocampal Neuroplasticity and Mitochondrial Function in Doxorubicin-Induced Chemobrain. *Neuropharmacology* **133**, 451-461 (2018).

76 Acheson, A. et al. A Bdnf Autocrine Loop in Adult Sensory Neurons Prevents Cell Death. *Nature* **374**, 450-453 (1995).

77 Huang, E. J. & Reichardt, L. F. Neurotrophins: Roles in Neuronal Development and Function. *Annu Rev Neurosci* **24**, 677-736 (2001).

78 Moore, I. M. et al. Effects of Intraventricular Methotrexate on Neuronal Injury and Gene Expression in a Rat Model: Findings from an Exploratory Study. *Biol Res Nurs* **18**, 505-514 (2016).

79 Gibson, E. M. et al. Methotrexate Chemotherapy Induces Persistent Tri-Glial Dysregulation That Underlies Chemotherapy-Related Cognitive Impairment. *Cell* **176**, 43-55 e13 (2019).

eFIGURES

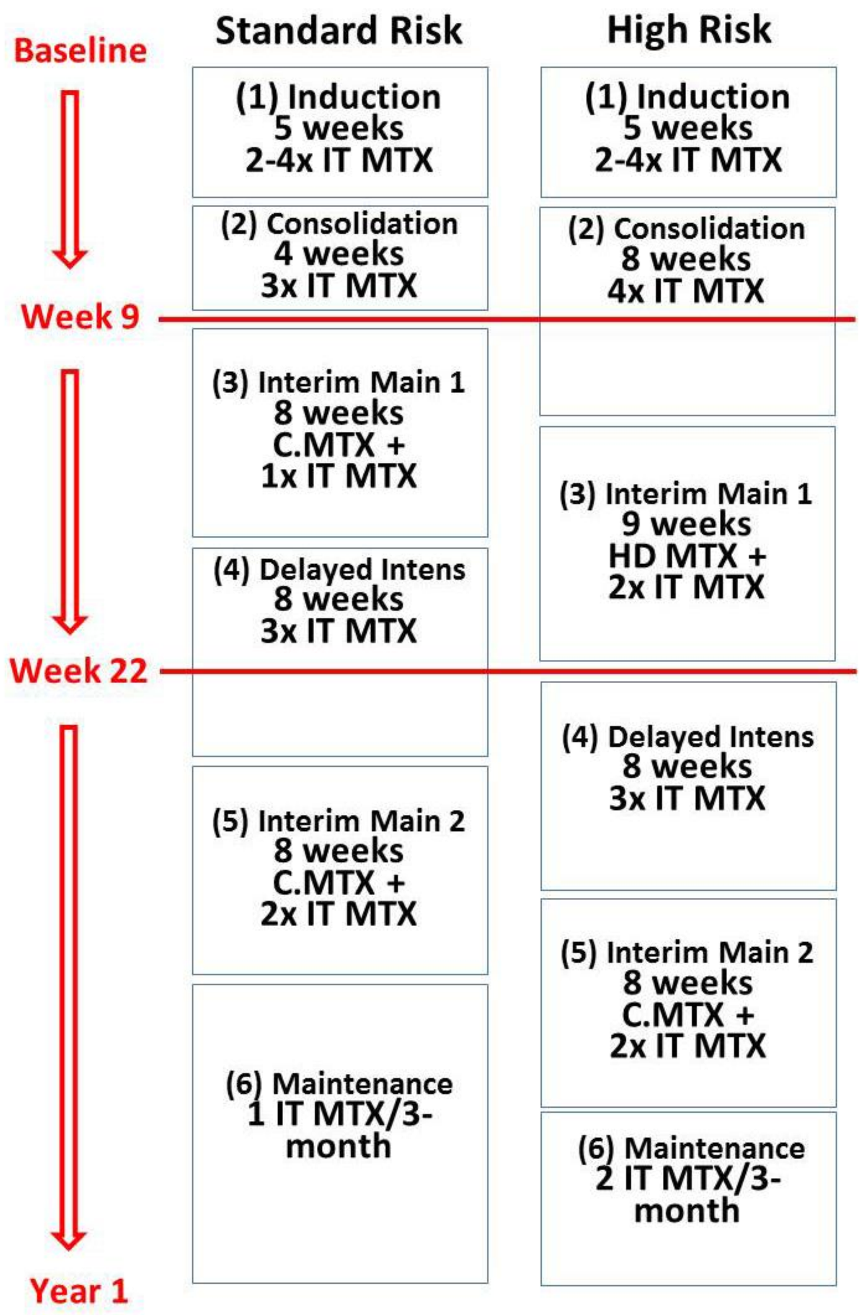

eFigure 1: Study Assessments and Children's Oncology Group Chemotherapy Treatment Protocol
Patients were treated with risk-stratified, standardized Children's Oncology Group regimens. MRI and neuropsychological assessments were collected at 4 timepoints before, during, and after chemotherapy. The assessment timepoints in relation for the chemotherapy are shown in red lines. Healthy controls were scanned at baseline and year 1.

MTX= Methotrexate; **IT MTX** = Intrathecal MTX; **HDMTX**=High dose MTX; **C.MTX**= Capizzi MTX (systemic MTX followed by L-asparaginase); **Intens**= intensification; **Maint**= maintenance.

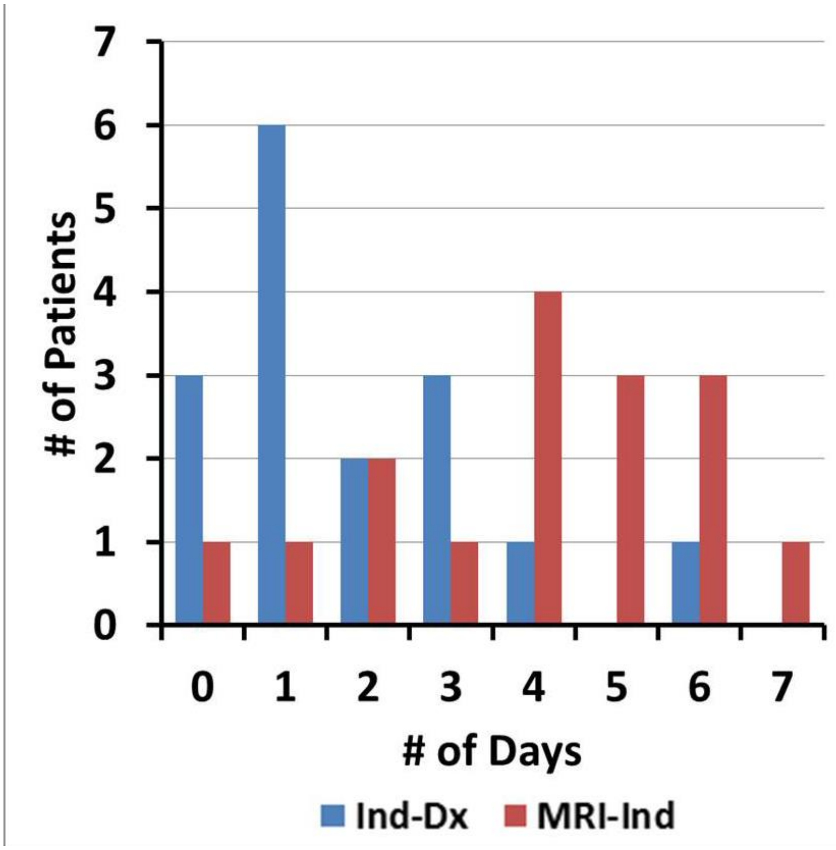

eFigure 2: Distribution of Days from initial diagnosis to chemotherapy initiation (*blue bars*) and from chemotherapy initiation to baseline assessments (*brown bars*) These distributions show that chemotherapy was started within 3 days of the initial diagnosis in almost all patients. Furthermore, pre-methotrexate baseline assessments were collected within 7 days of the initiation of chemotherapy, before patients received any doses of intrathecal or systemic methotrexate.

Ind-Dx=days from initial diagnosis to start of induction therapy; **MRI-Ind**=days from start of induction therapy to pre-methotrexate MRI

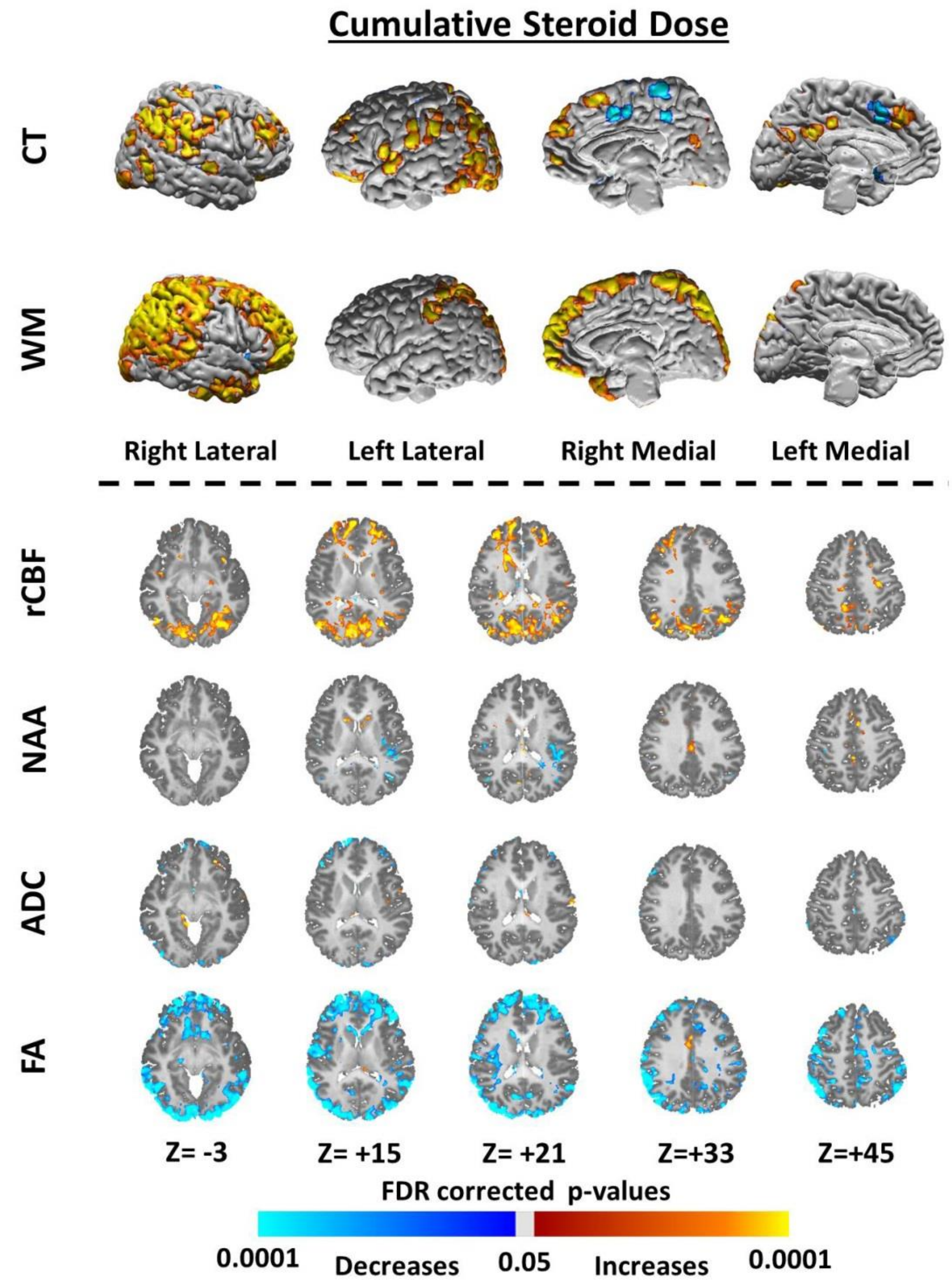

eFigure 3: Associations of Cumulative Corticosteroid Dose with Baseline Brain Measures We computed cumulative, equivalent doses of prednisone to dexamethasone received prior to the pre-MTX baseline MRI scan and applied multiple linear regression analyses to assess associations of cumulative steroid dose with baseline brain measures. We incorporated age as a covariate and employed the Topological FDR procedure to control for false positives in multiple hypotheses testing across the entire brain. Colors *violet* and *blue* show significant inverse, whereas colors *yellow* and *red* show significant positive, associations between cumulative corticosteroid dose and change in brain measures. The color bar at the bottom shows the color encoding of p-values. Corticosteroid dose was not associated with brain measures in WM regions that had abnormalities at baseline (**Fig.1**). In contrast, higher corticosteroid dose was associated with thicker cortical mantel but lower FA and higher rCBF values in the cortex.

CT=cortical thickness; **WM**=white matter; **rCBF**=regional cerebral blood flow; **NAA**=N-acetyl aspartate; **ADC**=average diffusivity coefficient; **FA**=fractional anisotropy; **MTX**=methotrexate

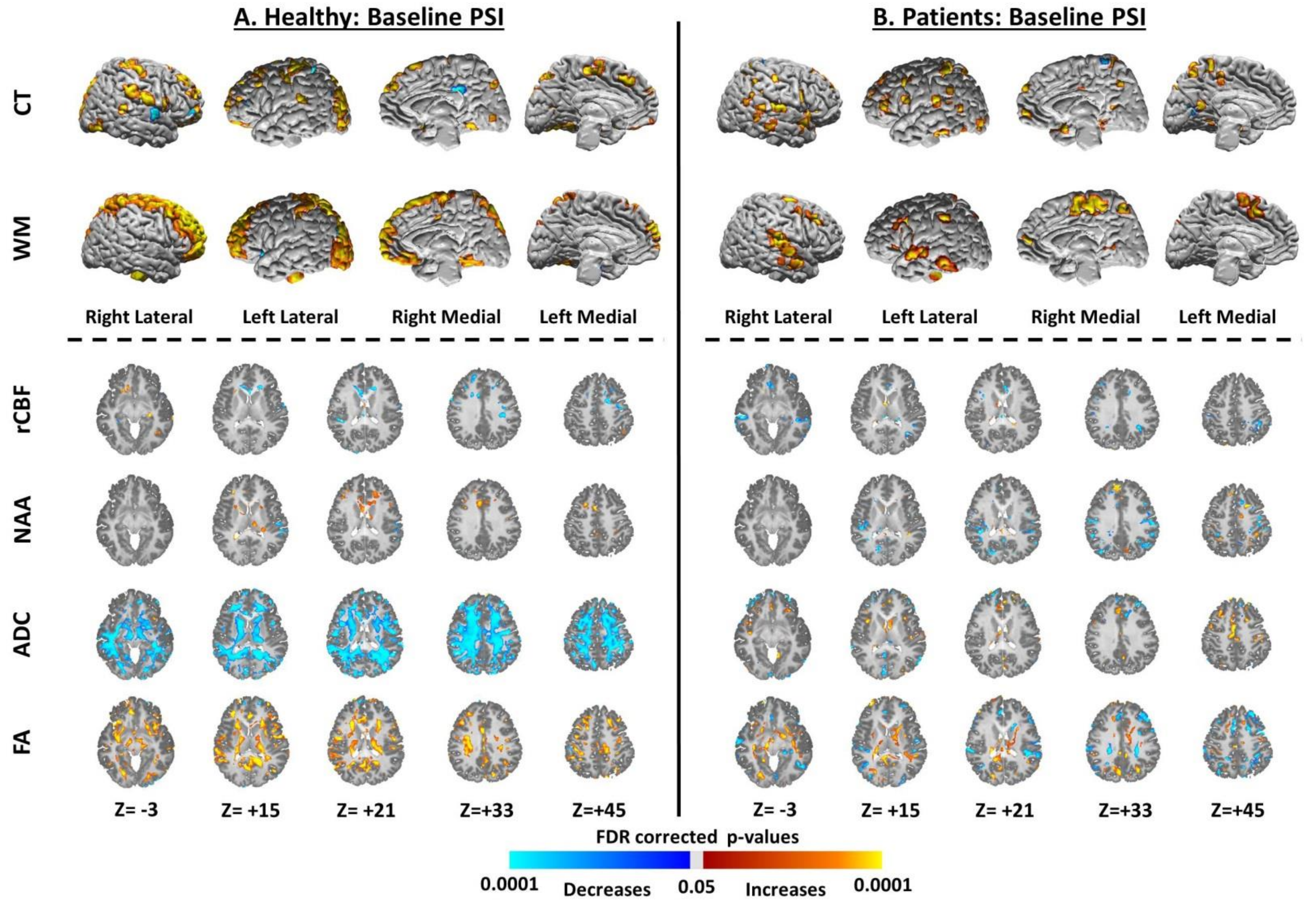

eFigure 4 A&B: Associations of Performance Speed Index (PSI) with Baseline Brain Measures We computed PSI using the scaled scores for the Coding and Symbol Search subtests and assessed its associations with brain measures at baseline, separately in healthy controls and patients as well as patients and healthy participants combined. We controlled for age and diagnosis by including them as covariates and controlled for false positives using the Topological FDR procedure in multiple linear regression analyses. P-values that survived the multiple comparisons procedure were color encoded and displayed on the template brain. Colors *violet* and *blue* show significant inverse, whereas colors *yellow* and *red* show significant positive, associations between cumulative corticosteroid dose and change in brain measures. The color bar at the bottom shows the color encoding of p-values.

CT=cortical thickness; **WM**=white matter; **rCBF**=regional cerebral blood flow; **NAA**=N-acetyl aspartate; **ADC**=average diffusivity coefficient; **FA**=fractional anisotropy; **PSI**=performance speed index.

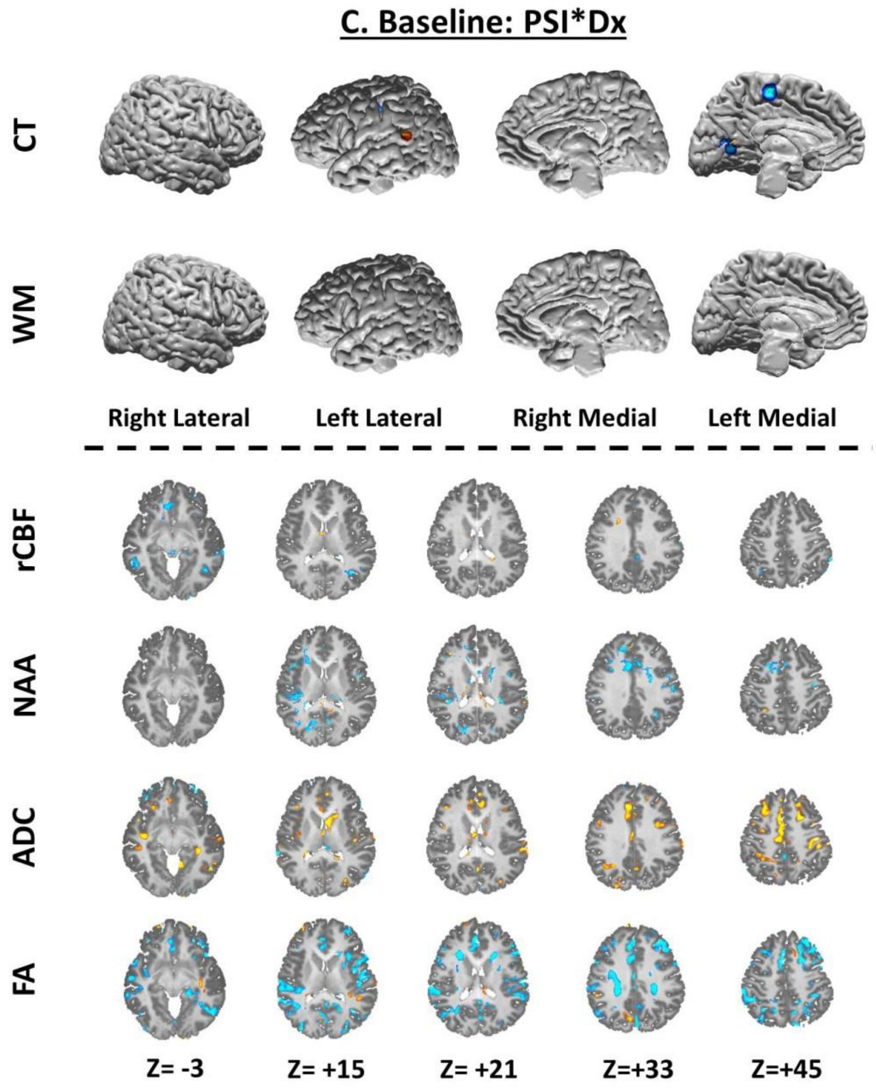

eFigure 4 C: Associations of Performance Speed Index (PSI) with Baseline Brain Measures

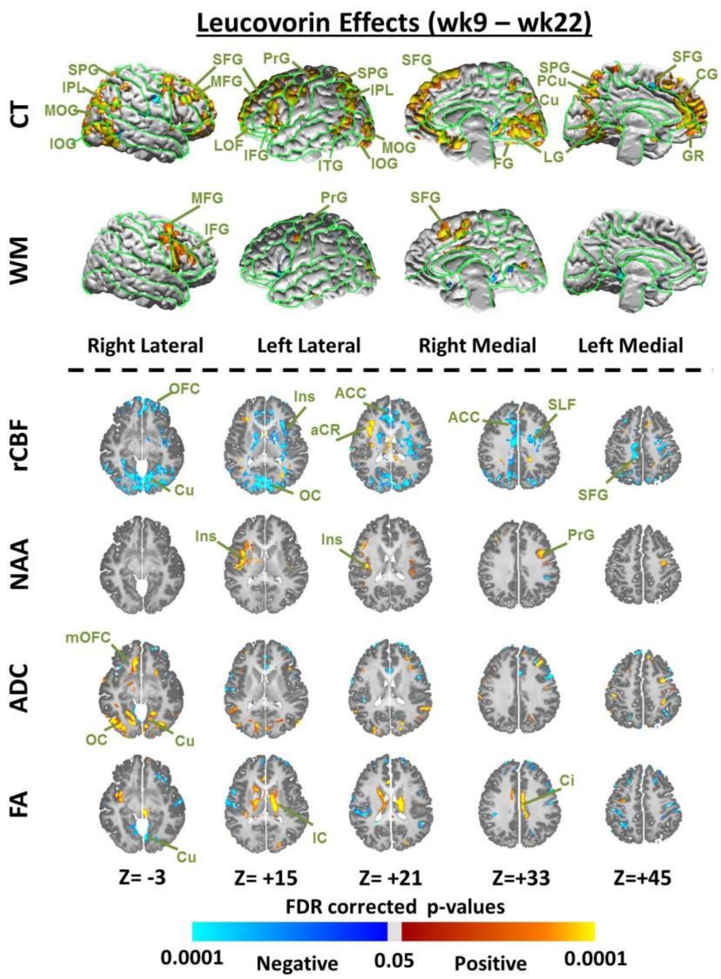

eFigure 5: Associations of Leucovorin Rescue with Changes in Brain Measures from week 9 to week 22 in patients with HR ALL. We conducted univariate repeated measures analyses using week 9 and week 22 data within patients with HR ALL to assess whether the use of leucovorin rescue was associated with the change in MRI-derived brain measures from week 9 to week 22. We controlled for age and IT-MTX effects on the brain. We controlled for false positives in multiple hypotheses testing using a procedure for Topological FDR; P-values that survived this procedure were color encoded and displayed either on the surface (rows *CT and WM*) or on the axial slices (rows *rCBF, NAA, ADC, FA*) of the template brain. Violet and blue show significant inverse whereas yellow and red show significant positive associations between leucovorin dose and change in brain measures. The color bar at the bottom shows the color encoding of p-values. Patients who received higher doses of leucovorin rescue had thicker cortex, greater WM local volumes in IFG and SFG, decreased rCBF in GM but increased rCBF in WM, increased NAA, increased ADC in GM, and increased FA in WM but decreased FA in GM.

CT=cortical thickness; **WM**=white matter; **rCBF**=regional cerebral blood flow; **NAA**=N-acetyl aspartate; **ADC**=average diffusivity coefficient; **FA**=fractional anisotropy;
SFG=superior frontal gyrus; **MFG**=middle frontal gyrus; **IFG**=inferior frontal gyrus; **DLPFC**= dorsolateral prefrontal cortex; **LOF**= lateral orbitofrontal gyrus; **MOF**= middle orbitofrontal gyrus; **SPG**=superior parietal gyrus; **MPG**=middle parietal gyrus; **STG**= superior temporal gyrus; **MTG**= middle temporal gyrus; **ITG**= inferior temporal gyrus; **MOG**= middle occipital gyrus; **IOG**= inferior occipital gyrus; **PoG**= postcentral gyrus; **PrG**= precentral gyrus; **CG**= cingulate gyrus; **dCG**= dorsal cingulate gyrus; **ACC**= anterior cingulate cortex; **PCC**= posterior cingulate cortex; **Tp**= Temporal pole; **Cu**= cuneus; **PCu**= precuneus; **LG**=lingual gyrus; **GR**= gyrus rectus; **FG**= fusiform gyrus; **CN**=Caudate Nucleus; **Put**= putamen; **Thal**= thalamus; **SLF**= superior longitudinal fasciculus; **gCC**= genu of corpus callosum; **sCC**= splenium of corpus callosum; **EC**= external

capsule; **IC**= internal capsule; **aCR**= anterior corona radiata; **sCR**= superior corona radiata; **pCR**= posterior corona radiata; **PTR**= posterior thalamic radiations; **OR**= optic radiations; **PTR**=Posterior Thalamic Radiations; **IFO**=Inferior Frontal-Occipital Fasciculus;

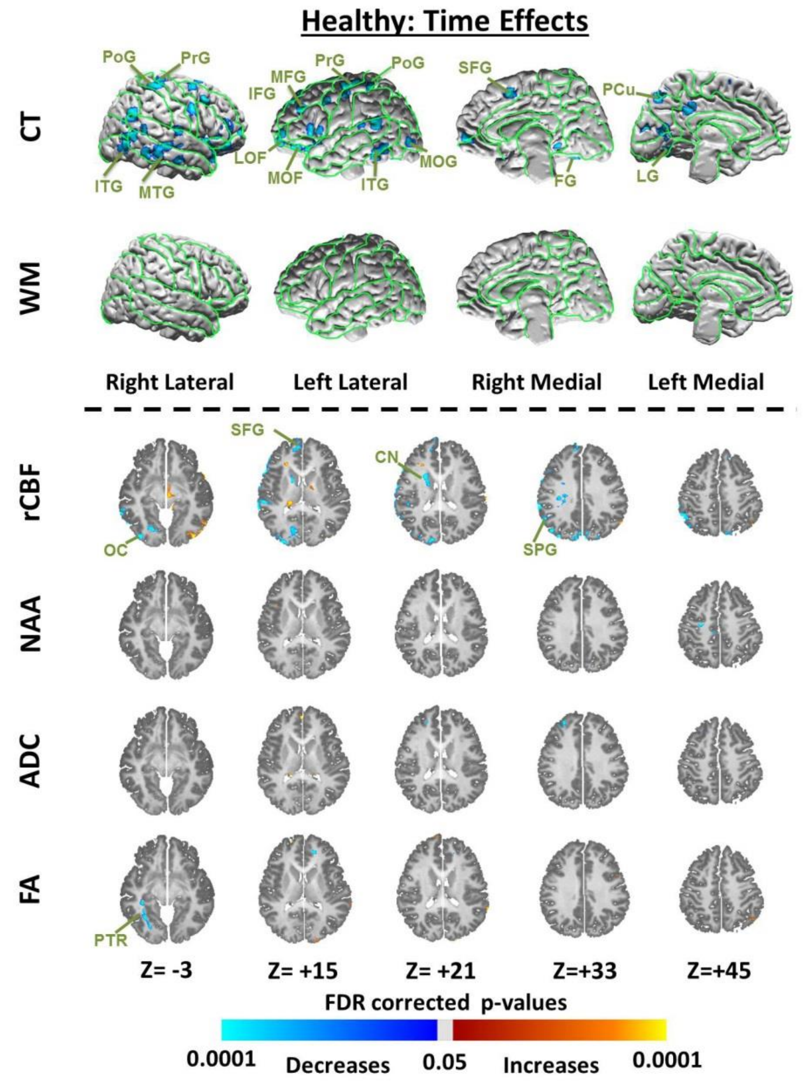

eFigure 6: Changes in MRI-Derived Brain Measures within Healthy Controls from baseline to year 1. We controlled for nuisance effects of age and computed the statistical significance of the time on the brain. We controlled for false positives in multiple hypotheses testing using a procedure for Topological FDR; P-values that survived this procedure were color encoded and displayed either on the surface (rows *CT and WM*) or on the axial slices (rows *rCBF*, *NAA*, *ADC*, *FA*) of the template brain. Violet and blue show significant decreases whereas yellow and red show significant increases in patients from baseline to year 1. The color bar at the bottom shows the color encoding of p-values. WM measures did not change with time. However, controls showed thinning of the cortex and decreases in rCBF values over the 1-year period.

CT=cortical thickness; **WM**=white matter; **rCBF**=regional cerebral blood flow; **NAA**=N-acetyl aspartate; **ADC**=average diffusivity coefficient; **FA**=fractional anisotropy;
SFG=superior frontal gyrus; **MFG**=middle frontal gyrus; **IFG**=inferior frontal gyrus; **DLPFC**= dorsolateral prefrontal cortex; **LOF**= lateral orbitofrontal gyrus; **MOF**= middle orbitofrontal gyrus; **SPG**=superior parietal gyrus; **MPG**=middle parietal gyrus; **STG**= superior temporal gyrus; **MTG**= middle temporal gyrus; **ITG**= inferior temporal gyrus; **MOG**= middle occipital gyrus; **IOG**= inferior occipital gyrus; **PoG**= postcentral gyrus; **PrG**= precentral gyrus; **CG**= cingulate gyrus; **dCG**= dorsal cingulate gyrus; **ACC**= anterior cingulate cortex; **PCC**= posterior cingulate cortex; **Tp**= Temporal pole; **Cu**= cuneus; **PCu**= precuneus; **LG**=lingual gyrus; **GR**= gyrus rectus; **FG**= fusiform gyrus; **CN**=Caudate Nucleus; **Put**= putamen; **Thal**= thalamus; **SLF**= superior longitudinal fasciculus; **gCC**= genu of corpus callosum; **sCC**= splenium of corpus callosum; **EC**= external capsule; **IC**= internal capsule; **aCR**= anterior corona radiata; **sCR**= superior corona radiata; **pCR**= posterior

corona radiata; **PTR**= posterior thalamic radiations; **OR**= optic radiations; **PTR**=Posterior Thalamic Radiations; **IFO**=Inferior Frontal-Occipital Fasciculus;

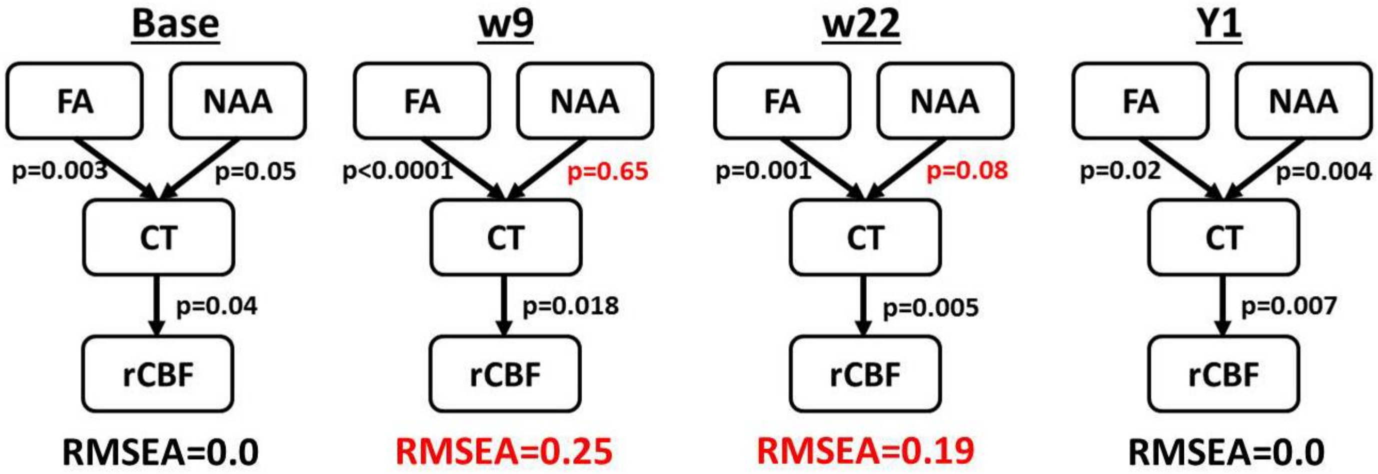

eFigure 7: Structural Equation Model (SEM) for Causal Relation among MRI-Derive Measures of brain structure (CT), microstructure (FA), metabolism (rCBF), and neuronal health (NAA). We conducted tertiary, exploratory analyses using MRI-derived measures averaged across brain regions that showed significant changes from baseline to year 1 of chemotherapy. We developed an SEM model for causal relations among brain measures, which tested whether neuronal integrity (NAA) or white matter integrity (FA) predicted cortical thickness (CT), which in turn predicted blood flow (rCBF). The p-values for predictive coefficients are shown along each arrow, with values in red showing predictive associations that did not reach statistical significance of 0.05. The fitted model accurately predicted population values at baseline. The predictive association between NAA and thickness was disrupted at week 9 and week 22, and therefore, the fitted model failed to accurately predict the population values. By year 1, the association between NAA and thickness was restored, and the fitted model predicted population values with high accuracy.

FA= fractional anisotropy; **CT**= cortical thickness; **NAA**= N-acetyl aspartate; **rCBF**= regional cerebral blood flow; **Base**=baseline; **w9**=week 9; **w22**= week 22; **Y1**=year 1; **RMSEA**=root mean square error of approximation

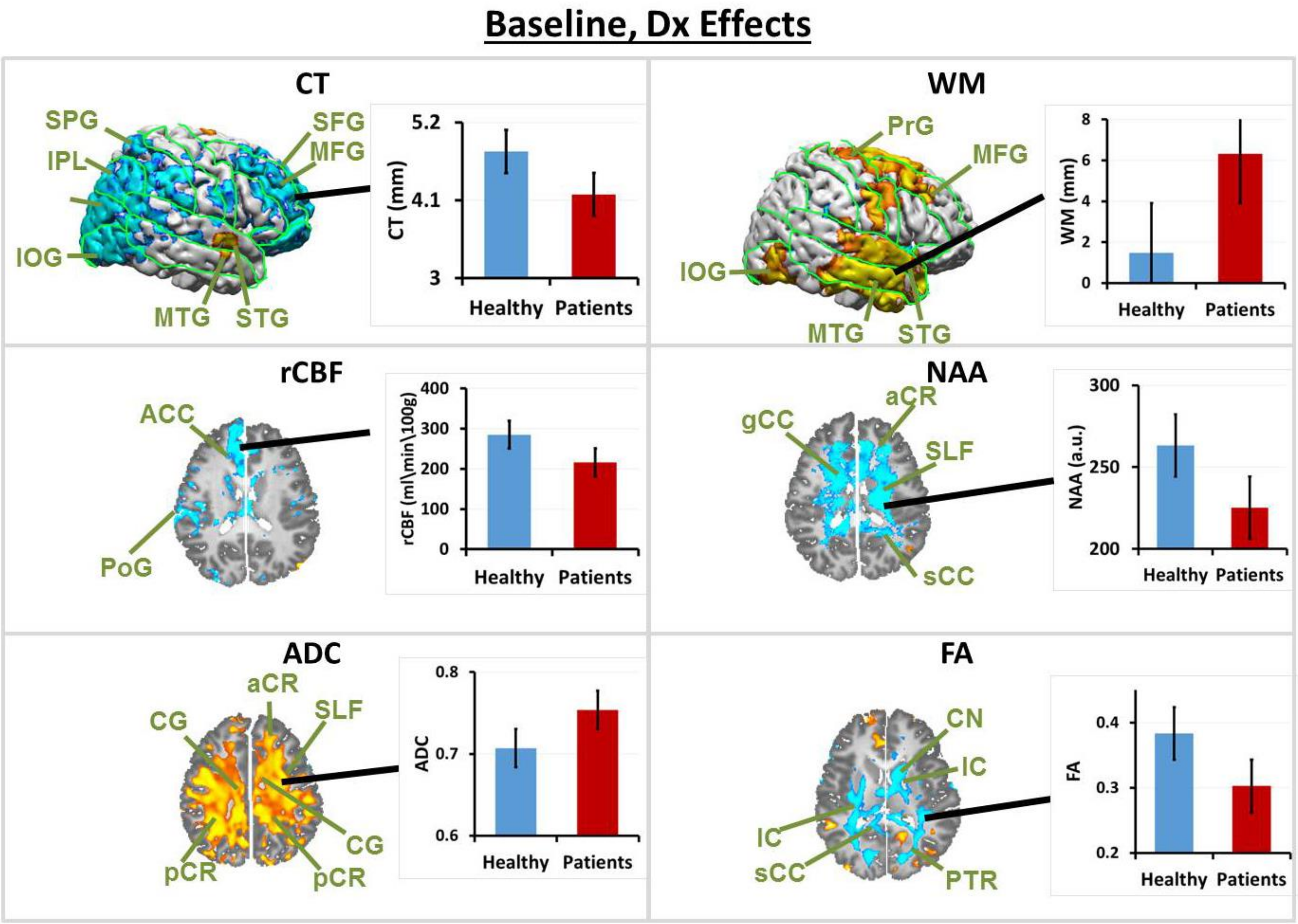

eFigure 8: Bar Graphs for Diagnosis Effects at Pre-Methotrexate Baseline We generated bar graphs for the various MRI-derived brain measures at a location indicated by the back line that differed significantly in patients compared to healthy controls (**Figure 1A**, main text). The table below presents the p-value, Student's t statistic, and effect size for the bar graphs shown in the regions of significant between-group differences.

	**p-value**	**t-statistic**	**Effect Size**
**CT**	**0.005**	**-2.97**	**0.84**
**WM**	**0.00048**	**4.8**	**1.33**
**rCBF**	**0.00017**	**-4.23**	**1.3**
**NAA**	**0.001**	**-3.57**	**1.41**
**ADC**	**0.0045**	**3.0**	**1.03**
**FA**	**0.013**	**-2.57**	**0.74**

Blue bars: data for healthy controls; **Red bars**: data for patients; **CT**=cortical thickness; **WM**=white matter; **rCBF**=regional cerebral blood flow; **NAA**=N-acetyl aspartate; **ADC**=average diffusivity coefficient; **FA**=fractional anisotropy;

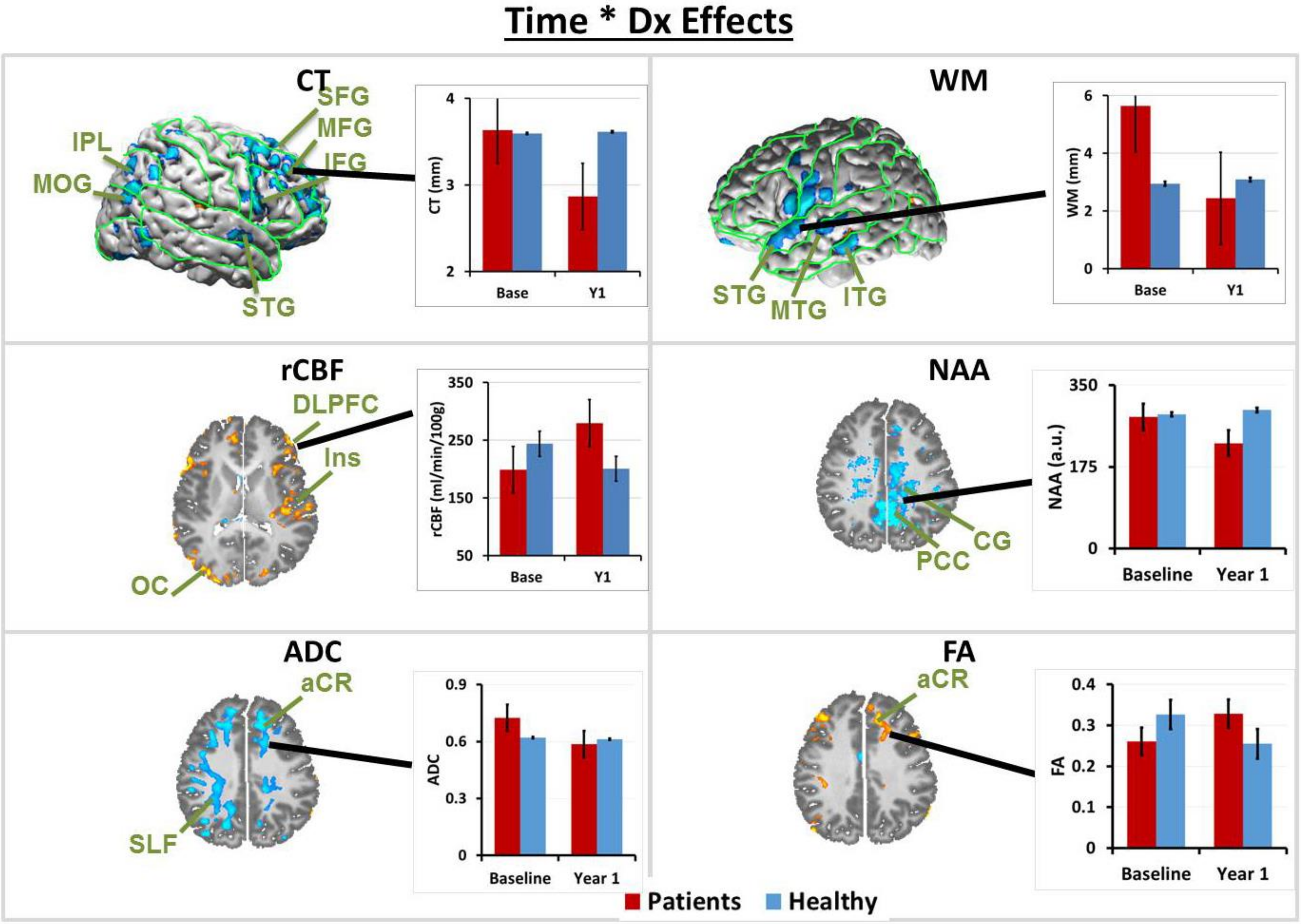

eFigure 9: Bar Graphs for Differing Time Effects in patients relative to healthy controls from baseline to year 1 assessments (**Figure 1B**, main text). The MRI data were sampled at a location indicated by the black lines. The table below presents the p-value and Student's t statistic for the time-by-Dx effects and R-square value for the fitted model in the regions of significant effects.

	p-value	t-statistic	R-Square
**CT**	**0.0013**	**3.36**	**0.46**
**WM**	**0.016**	**2.4**	**0.27**
**rCBF**	**0.002**	**3.24**	**0.16**
**NAA**	**0.004**	**3.0**	**0.27**
**ADC**	**0.015**	**2.5**	**0.30**
**FA**	**0.007**	**2.78**	**0.21**

Blue bars: data for healthy controls; **Red bars**: data for patients; **CT**=cortical thickness; **WM**=white matter; **rCBF**=regional cerebral blood flow; **NAA**=N-acetyl aspartate; **ADC**=average diffusivity coefficient; **FA**=fractional anisotropy;

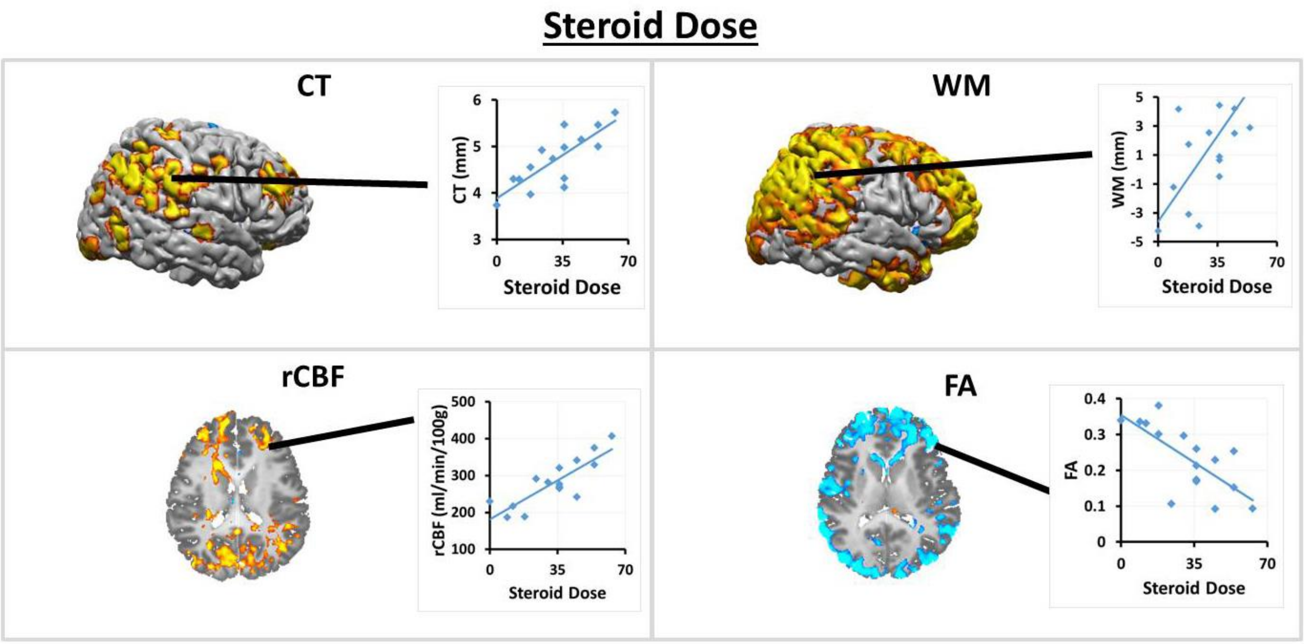

eFigure 10: Scatterplots for Associations of Cumulative Steroid Dose with brain measures at pre-methotrexate baseline. Steroid dose was significantly associated with cortical thickness, white matter volumes, rCBF, and fractional anisotropy (**eFigure 4**). The table below presents the p-value, Student's t statistic, and R-squared value for the fitted in the regions of significant steroid dose effects.

	**p-value**	**t-statistic**	**R-Square**
**CT**	**0.0006**	**4.47**	**0.82**
**WM**	**0.0034**	**3.5**	**0.51**
**rCBF**	**0.0003**	**4.96**	**0.69**
**FA**	**0.005**	**-3.32**	**0.61**

CT=cortical thickness; **WM**=white matter; **rCBF**=regional cerebral blood flow; **FA**=fractional anisotropy;

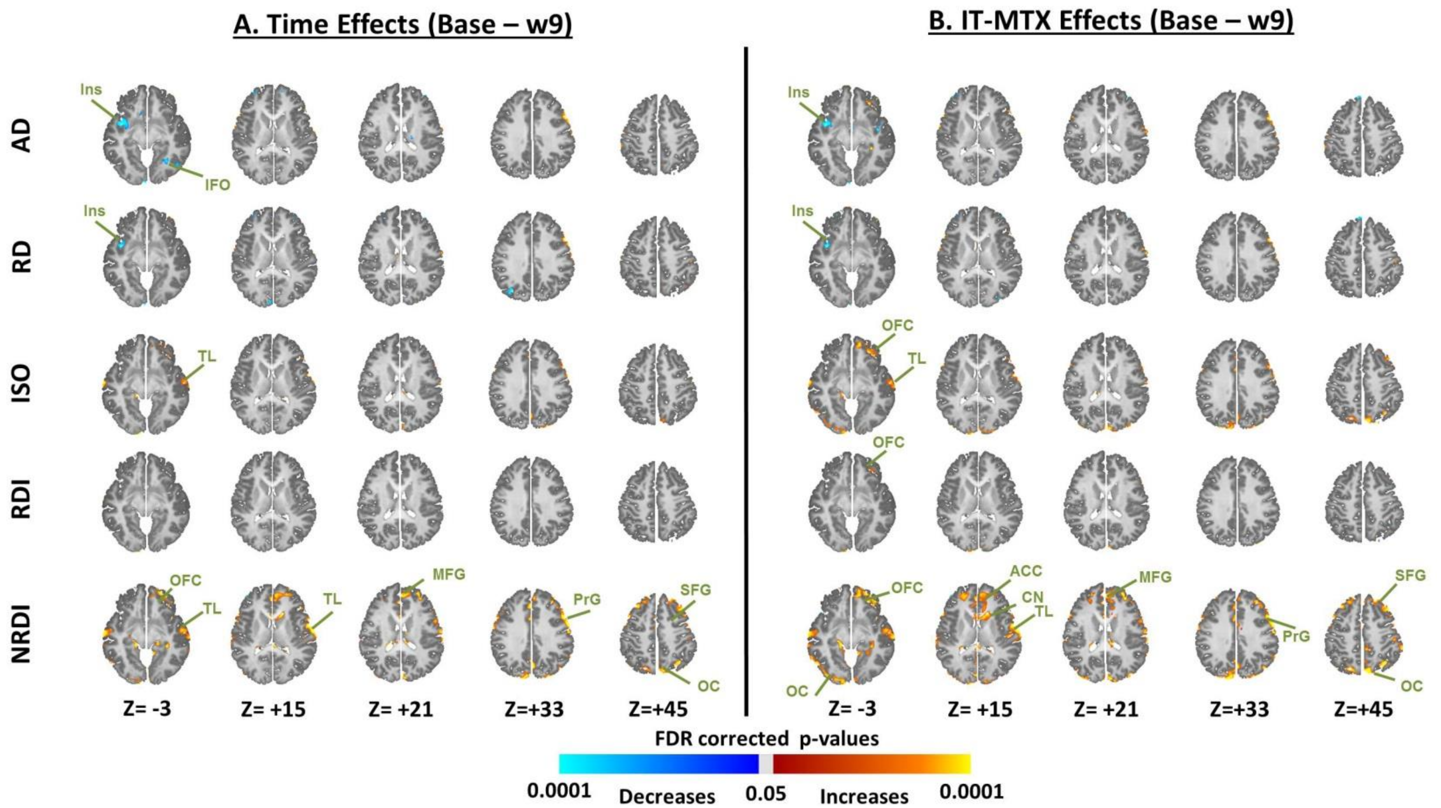

eFigure 11: Baseline to Week 9 changes in Multi-Shell DTI and QSI Brain Measures (*left panel*) and their Associations with IT-MTX Dose (*right panel*).

We conducted within-patient, univariate repeated measures analyses using baseline and week 9 data and assessed assess separately (1) how DTI and QSI measures changed from baseline to week 9 (*left panel*); and (2) whether those changes were associated with IT-MTX dose (*right panel*). We controlled for nuisance effects of age and risk of the time or the IT-MTX dose effects on the brain. We controlled for false positives in multiple hypotheses testing using a procedure for Topological FDR; P-values that survived this procedure were color encoded and displayed either on the axial slices of the template brain. Violet and blue show significant decreases whereas yellow and red show significant increases in patients from baseline to week 9. The color bar at the bottom shows the color encoding of p-values. DTI and QSI measures within WM did not change with time in patients. However, AD and RD decreased in insular cortex, and ISO and NRDI indices increased in GM of TL, OFC, MFG, SFG, PrG, and OC, suggesting increased edema in GM. These changes in GM were significantly associated with IT-MTX dose received by the patients.

DTI=Diffusion Tensor Imaging; **QSI**=Q-Space Imaging; **Base**=Baseline; **w9**=week9; **AD**=axial diffusivity; **RD**=radial diffusivity; **ISO**=Isotropic diffusion; **RDI**=restricted diffusion imaging; **NRDI**=non-restricted diffusion imaging; **SFG**=superior frontal gyrus; **MFG**=middle frontal gyrus; **TL**=temporal lobe; **OFC**= orbital frontal cortex; **OC**= Occipital cortex; **PrG**= precentral gyrus; **CN**=caudate nucleus; **Ins**=insular cortex;

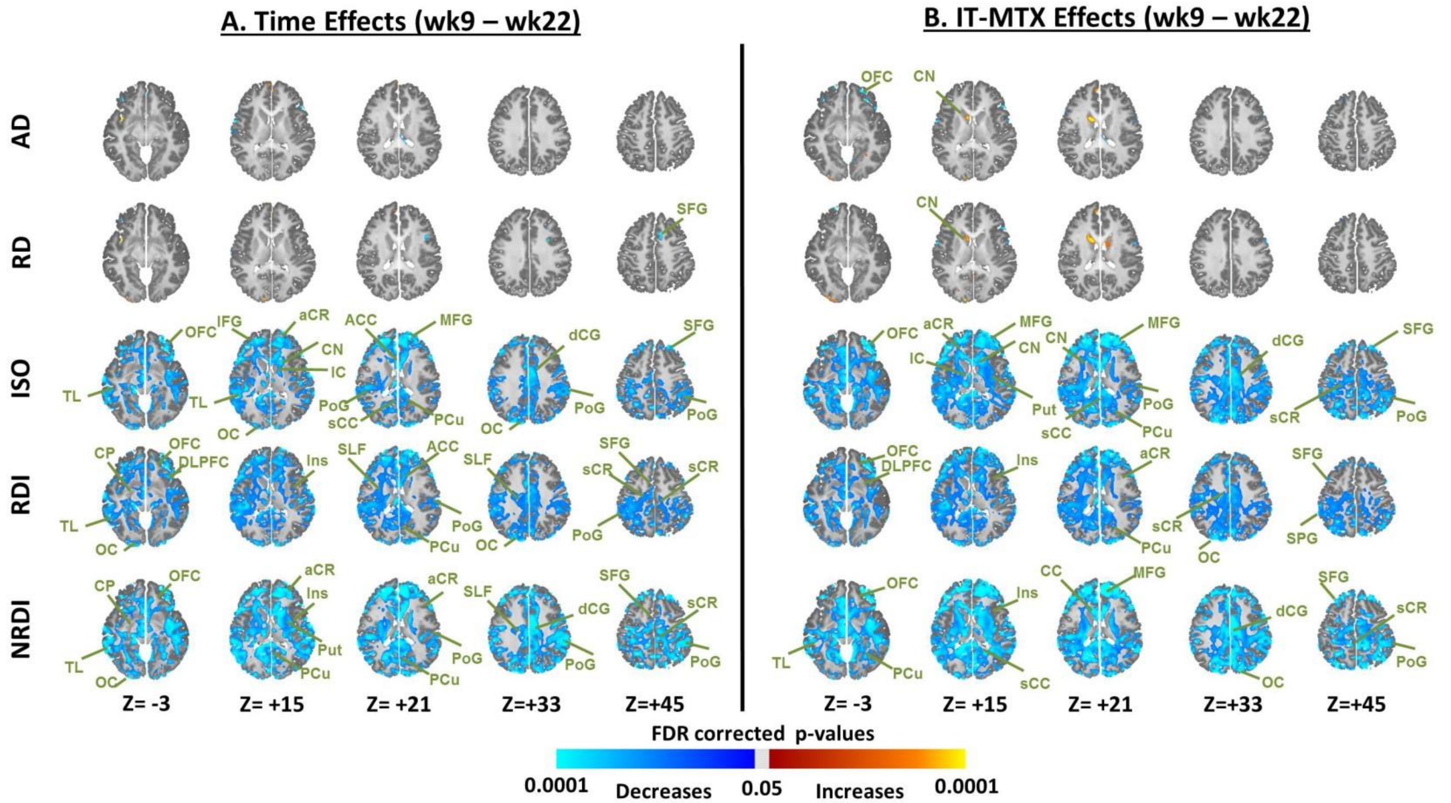

eFigure 12: Week 9 to Week 22 changes in Multi-Shell DTI and QSI Brain Measures (*left panel*) and their Associations with IT-MTX Dose (*right panel*)

We conducted within-patient, univariate repeated measures analyses using week 9 and week 22 data and assessed assess separately (1) how DTI and QSI measures changed from week 9 to week 22 (*left panel*); and (2) whether those changes were associated with IT-MTX dose (*right panel*). We controlled for nuisance effects of age and risk of the time or the IT-MTX dose effects on the brain. We controlled for false positives in multiple hypotheses testing using a procedure for Topological FDR; P-values that survived this procedure were color encoded and displayed either on the axial slices of the template brain. Violet and blue show significant decreases whereas yellow and red show significant increases in patients from baseline to week 9. The color bar at the bottom shows the color encoding of p-values. DTI measures of AD and RD largely did not change from week 9 to week 22. QSI measures of ISO, NRDI, and RDI however decreased with time, which were significantly associated with IT-MTX dose received by the patients.

DTI=Diffusion Tensor Imaging; **QSI**=Q-Space Imaging; **wk9**=week9; **wk22**=week22; **AD**=axial diffusivity; **RD**=radial diffusivity; **ISO**=Isotropic diffusion; **RDI**=restricted diffusion imaging; **NRDI**=non-restricted diffusion imaging;

SFG=superior frontal gyrus; **MFG**=middle frontal gyrus; **IFG**=inferior frontal gyrus; **DLPFC**= dorsolateral prefrontal cortex; **LOF**= lateral orbitofrontal gyrus; **MOF**= middle orbitofrontal gyrus; **SPG**=superior parietal gyrus; **MPG**=middle parietal gyrus; **STG**= superior temporal gyrus; **MTG**= middle temporal gyrus; **ITG**= inferior temporal gyrus; **MOG**= middle occipital gyrus; **IOG**= inferior occipital gyrus; **PoG**= postcentral gyrus; **PrG**= precentral gyrus; **CG**= cingulate gyrus; **dCG**= dorsal cingulate gyrus; **ACC**= anterior cingulate cortex; **PCC**= posterior cingulate cortex; **Tp**= Temporal pole; **Cu**= cuneus; **PCu**= precuneus; **LG**=lingual gyrus; **GR**= gyrus rectus; **FG**= fusiform gyrus; **CN**=Caudate Nucleus; **Put**= putamen; **Thal**= thalamus; **SLF**= superior longitudinal fasciculus; **gCC**= genu of corpus callosum; **sCC**= splenium of corpus callosum; **EC**= external capsule; **IC**= internal capsule; **aCR**= anterior corona radiata; **sCR**= superior corona radiata; **pCR**= posterior

corona radiata; **PTR**= posterior thalamic radiations; **OR**= optic radiations; **PTR**=Posterior Thalamic Radiations; **IFO**=Inferior Frontal-Occipital Fasciculus;

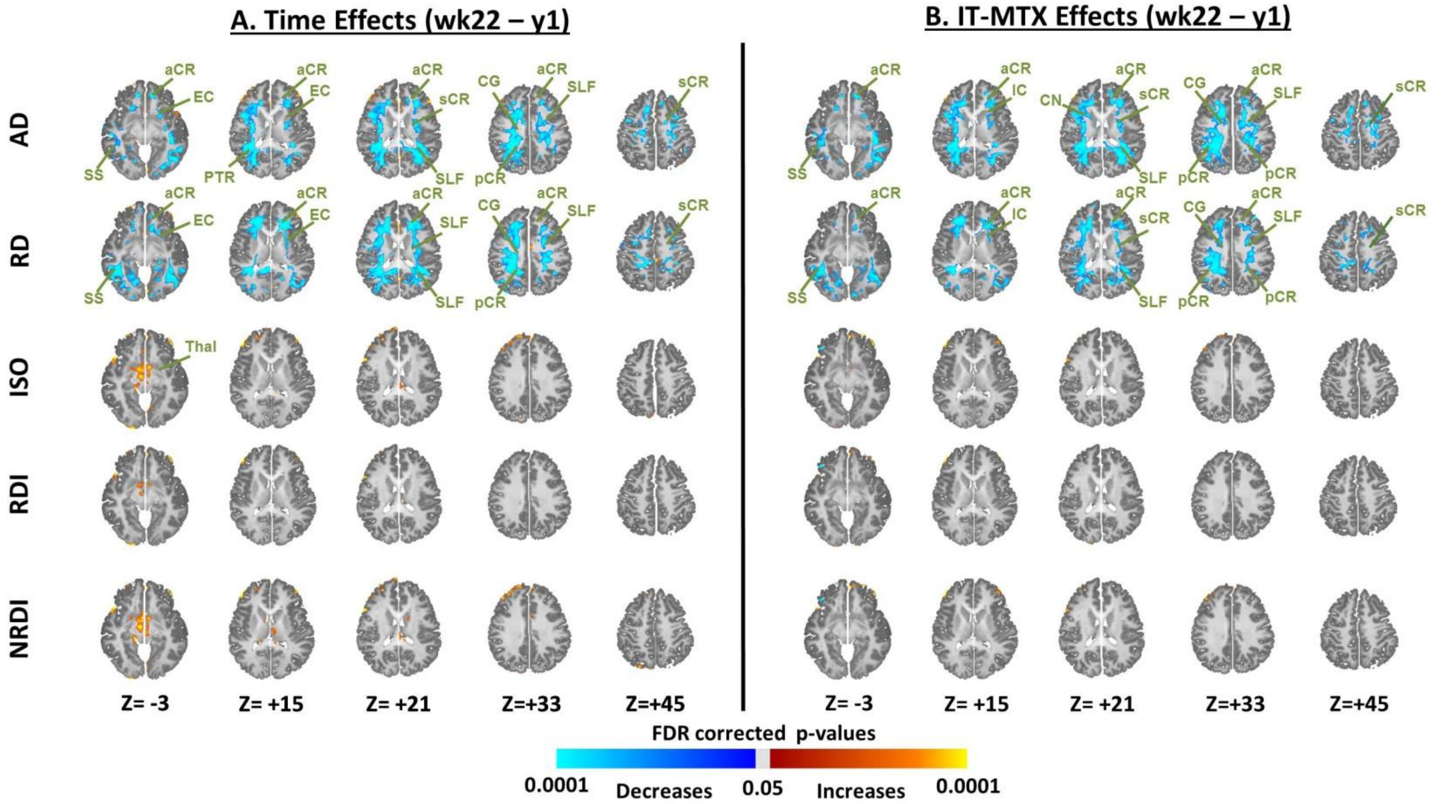

eFigure 13: Week 22 to Year 1 changes in Multi-Shell DTI and QSI Brain Measures (left panel) and their Associations with IT-MTX Dose (right panel)

We conducted within-patient, univariate repeated measures analyses using week 22 and year 1 data and assessed assess separately (1) how DTI and QSI measures changed from week 22 to year 1 (*left panel*); and (2) whether those changes were associated with IT-MTX dose (*right panel*). We controlled for nuisance effects of age and risk of the time or the IT-MTX dose effects on the brain. We controlled for false positives in multiple hypotheses testing using a procedure for Topological FDR; P-values that survived this procedure were color encoded and displayed either on the axial slices of the template brain. Violet and blue show significant decreases whereas yellow and red show significant increases in patients from baseline to week 9. The color bar at the bottom shows the color encoding of p-values. DTI measures of AD and RD decreased significantly within WM, and these changes were significantly associated with IT-MTX dose. In contrast, QSI measures of ISO, NRDI, and RDI largely did not change with time, except for small increases in thalamus.

DTI=Diffusion Tensor Imaging; **QSI**=Q-Space Imaging; **wk22**=Week 22; **y1**=year 1; **AD**=axial diffusivity; **RD**=radial diffusivity; **ISO**=Isotropic diffusion; **RDI**=restricted diffusion imaging; **NRDI**=non-restricted diffusion imaging;

SFG=superior frontal gyrus; **MFG**=middle frontal gyrus; **IFG**=inferior frontal gyrus; **DLPFC**= dorsolateral prefrontal cortex; **LOF**= lateral orbitofrontal gyrus; **MOF**= middle orbitofrontal gyrus; **SPG**=superior parietal gyrus; **MPG**=middle parietal gyrus; **STG**= superior temporal gyrus; **MTG**= middle temporal gyrus; **ITG**= inferior temporal gyrus; **MOG**= middle occipital gyrus; **IOG**= inferior occipital gyrus; **PoG**= postcentral gyrus; **PrG**= precentral gyrus; **CG**= cingulate gyrus; **dCG**= dorsal cingulate gyrus; **ACC**= anterior cingulate cortex; **PCC**= posterior cingulate cortex; **Tp**= Temporal pole; **Cu**= cuneus; **PCu**= precuneus; **LG**=lingual gyrus; **GR**= gyrus rectus; **FG**= fusiform gyrus; **CN**=Caudate Nucleus; **Put**= putamen; **Thal**= thalamus; **SLF**= superior longitudinal fasciculus; **gCC**= genu of corpus callosum; **sCC**= splenium of corpus callosum; **EC**= external capsule; **IC**= internal capsule; **aCR**= anterior corona radiata; **sCR**= superior corona radiata; **pCR**= posterior

corona radiata; **PTR**= posterior thalamic radiations; **OR**= optic radiations; **PTR**=Posterior Thalamic Radiations; **IFO**=Inferior Frontal-Occipital Fasciculus;

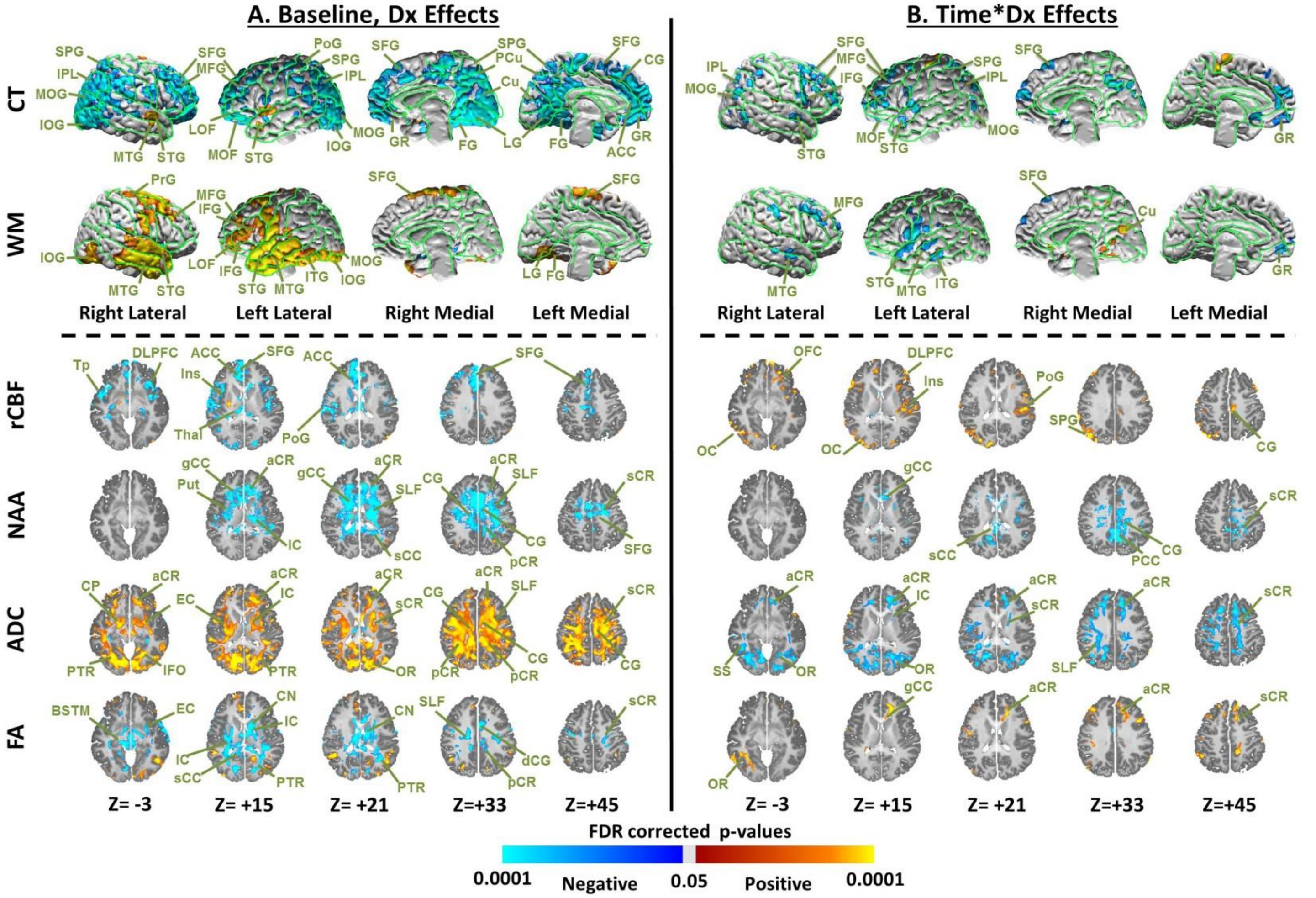

eFigure 14: Baseline Abnormalities and their Differential Progression in Patients relative to Healthy Controls We assessed (A) whether at pre-methotrexate baseline brain measures in patients differed from those in healthy controls (Dx Effects), and (B) whether brain measures changed differentially in patients relative to healthy controls (Time*Dx effects). Patients had significant GM and WM abnormalities at baseline that progressed differentially in patients. These enlarged images in **Figure 2, A & B** for the main text are presented here for clarity and visual localization of the findings.

Dx=diagnosis; **CT**=cortical thickness; **WM**=white matter; **rCBF**=regional cerebral blood flow; **NAA**=N-acetyl aspartate; **ADC**=average diffusivity coefficient; **FA**=fractional anisotropy;
SFG=superior frontal gyrus; **MFG**=middle frontal gyrus; **IFG**=inferior frontal gyrus; **DLPFC**= dorsolateral prefrontal cortex; **LOF**= lateral orbitofrontal gyrus; **MOF**= middle orbitofrontal gyrus; **SPG**=superior parietal gyrus; **MPG**=middle parietal gyrus; **STG**= superior temporal gyrus; **MTG**= middle temporal gyrus; **ITG**= inferior temporal gyrus; **MOG**= middle occipital gyrus; **IOG**= inferior occipital gyrus; **PoG**= postcentral gyrus; **PrG**= precentral gyrus; **CG**= cingulate gyrus; **dCG**= dorsal cingulate gyrus; **ACC**= anterior cingulate cortex; **PCC**= posterior cingulate cortex; **Tp**= Temporal pole; **Cu**= cuneus; **PCu**= precuneus; **LG**=lingual gyrus; **GR**= gyrus rectus; **FG**= fusiform gyrus; **CN**=Caudate Nucleus; **Put**= putamen; **Thal**= thalamus; **SLF**= superior longitudinal fasciculus; **gCC**= genu of corpus callosum; **sCC**= splenium of corpus callosum; **EC**= external capsule; **IC**= internal capsule; **aCR**= anterior corona radiata; **sCR**= superior corona radiata; **pCR**= posterior corona radiata; **PTR**= posterior thalamic radiations; **OR**= optic radiations; **PTR**=Posterior Thalamic Radiations; **IFO**=Inferior Frontal-Occipital Fasciculus;

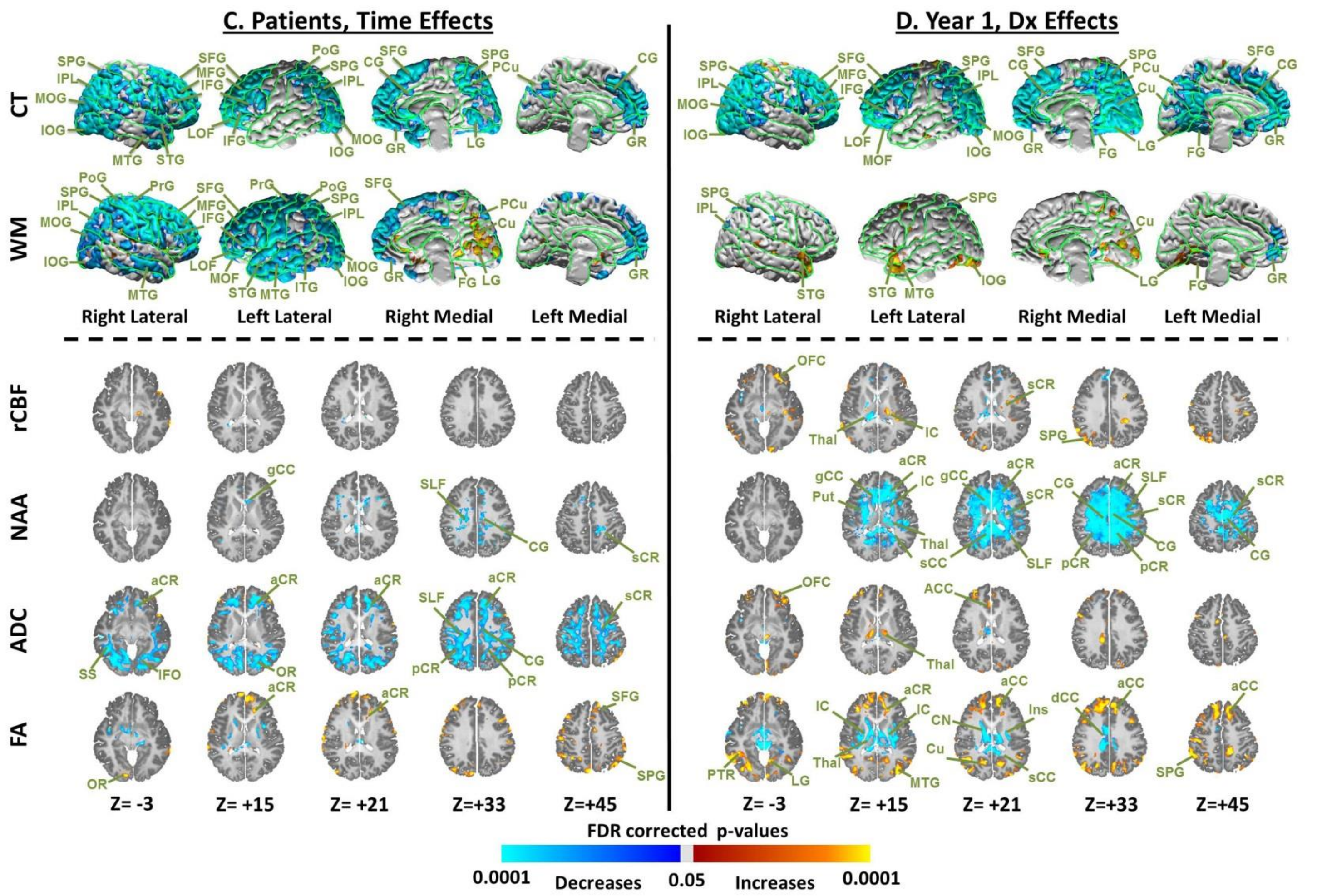

eFigure 15: Progression and Persistence of Abnormalities in Patients We assessed (C) how brain measures changed within patients alone (Time effects), and (D) whether patients had persisting brain abnormalities at year 1. GM abnormalities worsened but WM abnormalities normalized by year 1. These enlarged images in **Figure 2, C & D** for the main text are presented here for clarity and visual localization of the findings.

Dx=diagnosis; **CT**=cortical thickness; **WM**=white matter; **rCBF**=regional cerebral blood flow; **NAA**=N-acetyl aspartate; **ADC**=average diffusivity coefficient; **FA**=fractional anisotropy;
SFG=superior frontal gyrus; **MFG**=middle frontal gyrus; **IFG**=inferior frontal gyrus; **DLPFC**= dorsolateral prefrontal cortex; **LOF**= lateral orbitofrontal gyrus; **MOF**= middle orbitofrontal gyrus; **SPG**=superior parietal gyrus; **MPG**=middle parietal gyrus; **STG**= superior temporal gyrus; **MTG**= middle temporal gyrus; **ITG**= inferior temporal gyrus; **MOG**= middle occipital gyrus; **IOG**= inferior occipital gyrus; **PoG**= postcentral gyrus; **PrG**= precentral gyrus; **CG**= cingulate gyrus; **dCG**= dorsal cingulate gyrus; **ACC**= anterior cingulate cortex; **PCC**= posterior cingulate cortex; **Tp**= Temporal pole; **Cu**= cuneus; **PCu**= precuneus; **LG**=lingual gyrus; **GR**= gyrus rectus; **FG**= fusiform gyrus; **CN**=Caudate Nucleus; **Put**= putamen; **Thal**= thalamus; **SLF**= superior longitudinal fasciculus; **gCC**= genu of corpus callosum; **sCC**= splenium of corpus callosum; **EC**= external capsule; **IC**= internal capsule; **aCR**= anterior corona radiata; **sCR**= superior corona radiata; **pCR**= posterior corona radiata; **PTR**= posterior thalamic radiations; **OR**= optic radiations; **PTR**=Posterior Thalamic Radiations; **IFO**=Inferior Frontal-Occipital Fasciculus;
